# Different Mutagenic Potential of HIV-1 Restriction Factors APOBEC3G and APOBEC3F Is Determined by Distinct Single-Stranded DNA Scanning Mechanisms

**DOI:** 10.1371/journal.ppat.1004024

**Published:** 2014-03-20

**Authors:** Anjuman Ara, Robin P. Love, Linda Chelico

**Affiliations:** Department of Microbiology & Immunology, University of Saskatchewan, Saskatoon, Saskatchewan, Canada; Vanderbilt University School of Medicine, United States of America

## Abstract

The APOBEC3 deoxycytidine deaminase family functions as host restriction factors that can block replication of Vif (virus infectivity factor) deficient HIV-1 virions to differing degrees by deaminating cytosines to uracils in single-stranded (−)HIV-1 DNA. Upon replication of the (−)DNA to (+)DNA, the HIV-1 reverse transcriptase incorporates adenines opposite the uracils, thereby inducing C/G→T/A mutations that can functionally inactivate HIV-1. Although both APOBEC3F and APOBEC3G are expressed in cell types HIV-1 infects and are suppressed by Vif, there has been no prior biochemical analysis of APOBEC3F, in contrast to APOBEC3G. Using synthetic DNA substrates, we characterized APOBEC3F and found that similar to APOBEC3G; it is a processive enzyme and can deaminate at least two cytosines in a single enzyme-substrate encounter. However, APOBEC3F scanning movement is distinct from APOBEC3G, and relies on jumping rather than both jumping and sliding. APOBEC3F jumping movements were also different from APOBEC3G. The lack of sliding movement from APOBEC3F is due to an ^190^NPM^192^ motif, since insertion of this motif into APOBEC3G decreases its sliding movements. The APOBEC3G NPM mutant induced significantly less mutations in comparison to wild-type APOBEC3G in an *in vitro* model HIV-1 replication assay and single-cycle infectivity assay, indicating that differences in DNA scanning were relevant to restriction of HIV-1. Conversely, mutation of the APOBEC3F ^191^Pro to ^191^Gly enables APOBEC3F sliding movements to occur. Although APOBEC3F ^190^NGM^192^ could slide, the enzyme did not induce more mutagenesis than wild-type APOBEC3F, demonstrating that the unique jumping mechanism of APOBEC3F abrogates the influence of sliding on mutagenesis. Overall, we demonstrate key differences in the impact of APOBEC3F- and APOBEC3G-induced mutagenesis on HIV-1 that supports a model in which both the processive DNA scanning mechanism and preferred deamination motif (APOBEC3F, 5′TTC; APOBEC3G 5′CCC) influences the mutagenic and gene inactivation potential of an APOBEC3 enzyme.

## Author Summary

Human cells possess a family of seven DNA-modification enzymes, termed APOBEC3, that function as part of our innate immune system. The enzymes modify cytosine in DNA which induces mutations. There are particular enzymes, APOBEC3D, APOBEC3F, APOBEC3G and APOBEC3H, that appear to be most relevant to restricting HIV-1 replication in CD4+ T cells using this mutagenic mechanism, if they can avoid degradation that is induced by the HIV-1 protein Vif. There has been little biochemical analysis of APOBEC3 enzymes other than APOBEC3G in terms of the mechanism by which these enzymes search DNA for target cytosines to deaminate. We conducted a biochemical analysis of APOBEC3F. We found that while APOBEC3G uses 1-dimensional sliding and 3-dimensional translocations, APOBEC3F is restricted to 3-dimensional translocations. This makes the searching mechanism of APOBEC3F superficial and detrimental to the induction of a large number of mutations. In addition, gene inactivation was less likely to occur upon deamination of the target motif of APOBEC3F (5′TTC), in comparison to the target motif of APOBEC3G (5′CCC). All together the data support a model in which the way these enzymes scan DNA can predict the magnitude of mutagenesis induced and the target motif can predict ability to cause gene inactivation.

## Introduction

APOBEC3F (A3F) and APOBEC3G (A3G) are members of a family of seven single-stranded (ss)DNA cytosine deaminases (A3A, A3B, A3C, A3D, A3F, A3G, and A3H) [1] and play a role in restriction of the retrovirus HIV-1 (referred to as HIV) [2]. Research has been highly focused on primarily A3G and secondarily A3F for a number of years since they appeared to be the most efficient restrictors of HIV replication [3], [4], [5], [6], [7]. Although there are documented restrictive effects of A3G, and possibly A3F, at an individual level (reviewed in [8]), the suppression of HIV by A3G and A3F at a population level is lost due to the HIV protein Vif (viral infectivity factor) [6], [9]. Vif forms an E3 ubiquitin ligase with host proteins and causes A3G and A3F polyubiquitination and degradation through the proteasome [6], [10], [11], [12], [13], [14].

The general mechanism by which A3G restricts HIV, which has been a paradigm for other A3 enzymes, requires that it be encapsidated with the ribonucleoprotein complex of HIV [9], [15]. A3G requires its N-terminal domain (NTD), which can bind nucleic acids, for encapsidation into virions [16]. A3G catalyzes deaminations through its C-terminal deaminase domain (CTD) [16], [17]. In the target cells that these virions infect, encapsidated A3G can deaminate cytosines to uracils in (−)DNA reverse transcribed from the RNA genome, after the reverse transcriptase associated RNaseH activity enables ssDNA regions on the (−)DNA to be accessed by the enzyme [18], [19]. The uracils in the (−)DNA are used as a template by reverse transcriptase during (+)DNA synthesis and result in guanine to adenine mutations. If A3G can induce sufficient numbers of these mutations, the resulting proviral DNA will be functionally inactivated. The deaminases A3D, A3F, and A3H appear to follow this general mechanism of restriction in cell culture, but to differing degrees than A3G [3], [4], [20], [21], [22], [23], [24]. The exceptions are A3A, which inhibits incoming HIV viral particles in myeloid lineage cells [25], [26], A3C, which does not appear to become encapsidated or restrict HIV in cell culture [20], [27], and A3B, which can restrict HIV in 293T and HeLa cells, but not SupT1 cells [20], [28].

Despite a possible role for A3F, A3D and A3H Haplotype II in HIV restriction, it appears that A3G is more effective at restricting HIV replication and that perhaps the other A3 enzymes function in a collaborative way with A3G [20], [21], [22], [23], [24], [29]. In particular there has been a recent focus on the restriction capability of A3F. A3F was initially identified as potentially being an equal contributor with A3G to the restriction of HIV [3], [5], [6], [7], [30], but current research demonstrates, in agreement with an earlier report [4], that A3F may have less antiviral activity than A3G [22], [23], [24], [29]. Many different experimental protocols, such as analysis of stably expressed A3F from a cell line [23], use of primary cell lines [24], and A3F haplotypes from donor samples [22] have been applied and demonstrate that A3F has less of an effect on HIV infectivity in comparison to A3G. However, another report showed no difference in restriction efficiency of A3G and A3F beyond 2-fold using experiments that knocked-down endogenous A3 expression in a nonpermissive cell line [21]. As a result, the role of A3F in restriction of HIV remains unclear.

Among reports demonstrating less of an affect of A3F in restricting HIV replication than A3G, there is still no identified reason for why this may occur. From some reports A3F mRNA is expressed 10-fold [31] or 5-fold [22] less than A3G mRNA, suggesting less A3F would become virion encapsidated. However another report found A3F and A3G mRNA expression was more comparable [32]. Further, some reports have found a direct correlation with mRNA and protein levels [31], [32] whereas other reports have been unable to make such a correlation due to the use of different primary antibodies [24]. Confounding the interpretation of these data are reports which demonstrated that A3F is preferentially encapsidated with the HIV ribonucleoprotein complex in comparison to A3G [4], [33]. Song *et al.* concluded that the encapsidation difference between A3G and A3F in effect absolves any difference in cellular expression [33]. Despite this observed more specific packaging of A3F in the ribonucleoprotein complex [33], studies have found a minimal contribution of A3F to the hypermutation of HIV genomes or less potency in HIV-1 restriction [4], [22], [23], [24], [29]. Together these data suggest that if there is a difference in restriction efficiency of A3F and A3G, that it is not the physiological conditions which cause different effects on HIV infectivity, but an inherent difference in their biochemical characteristics. However, there has been no in depth biochemical characterization of A3F to date to determine what might be these differences between A3G and A3F. As such, we have undertaken a characterization of A3F in comparison to A3G to identify an underlying biochemical reason for these observations.

In particular, we have focused on characterizing the mechanism A3F uses to scan ssDNA. This is because it has been shown that the ssDNA scanning mechanism of A3G is important for inducing mutagenesis of (−)DNA formed during reverse transcription of RNA [34]. A3G has been characterized to scan ssDNA through facilitated diffusion [35], [36], [37]. Facilitated diffusion is a 3-dimensional scan of DNA by enzymes to locate their target sites for catalysis [38], [39], [40]. The movement is characterized by sliding, jumping or intersegmental transfer motions. Sliding is used to describe short range 1-dimensional scanning motions and can enable an in depth search of a particular area of DNA for a target motif [39], [40]. Jumping is a term that describes microdissociations of the enzyme from the DNA with a reassociation on the same DNA substrate, i.e., the enzyme does not diffuse into the bulk solution [38], [41]. The negative charge of the DNA establishes a charged radius around the DNA molecule in which a positively charged enzyme can dissociate, diffuse and still return back to the same DNA. These jumping events enable enzymes to translocate larger distances than sliding thus making the search of non-target DNA more efficient than sliding alone [39], [40]. Intersegmental transfer is similar to jumping but describes a movement where an enzyme with two DNA binding domains interacts with two distal sites simultaneously before dissociating from one of the sites [39], [40]. Different research groups, including our own, have found A3G to use a combined sliding and jumping search mechanism [34], [35], [37], [42], although one report found A3G to use intersegmental transfer [36]. We have characterized A3G mutants and A3G in complex with different Vif variants that resulted in decreases of either sliding or jumping motions and found that the ability of these A3G forms to induce mutagenesis of nascently reverse transcribed DNA was decreased [34], [43]. We have hypothesized that both sliding and jumping are important for inducing mutagenesis because A3G needs to conduct local searches (sliding) to effectively deaminate many cytosines, ensuring gene inactivation, and also translocate (jumping) over RNA/DNA hybrids to reach distal regions of (−)DNA [34]. The processive scanning of other A3 enzymes has not been reported, except A3A which was found to be largely nonprocessive [44].

This work is the first biochemical characterization of A3F and provides a biochemical explanation for the lowered ability of A3F to inactivate HIV, as reported by numerous research groups [22], [23], [24], [29] and within this report. We have found that A3F primarily uses jumping movements to scan ssDNA which is detrimental to its ability to cause numerous mutations on (−)DNA during reverse transcription. The target motif of A3G (5′CCC) also appears to cause more inactivating mutations in the HIV *protease* (*prot*) than the target motif for A3F (5′TTC), adding another level of deficiency in HIV inactivation potential. All together our data provide a model for the specific biochemical properties required for efficient restriction of HIV by A3 deaminases.

## Results

### A3F and A3G distinctively scan ssDNA

The processive nature of A3G has been shown to be of importance for inducing mutagenesis of HIV (−)DNA in a model *in vitro* system [34], [43] and in cell culture [45]. It is not known whether A3F is processive. Since multiple lines of evidence from independent labs have shown that the effect of A3F on HIV is different than A3G [4], [22], [23], [24], we sought to determine if there was an inherent biochemical difference between the two enzymes that could account for these observations. Specifically we determined if there was a difference in the processive scanning mechanisms of these two enzymes with processivity being defined as the ability to deaminate more than one cytosine on an ssDNA in a single-enzyme substrate encounter. Processivity was determined using different synthetic ssDNA substrates containing two deamination motifs separated by different distances, 5′TTC for A3F and 5′CCC for A3G. This strategy was used since with A3G we have found that closely spaced deamination motifs, i.e., 5 to 15 nt are deaminated most efficiently through sliding motions and as the distance between deamination motifs increases a jumping motion facilitates processive deaminations [34]. The substrate usage was kept below 15% to ensure single-hit conditions were maintained, which means that each ssDNA was only encountered by an enzyme at most once during the reaction [46].

**Figure 1:**
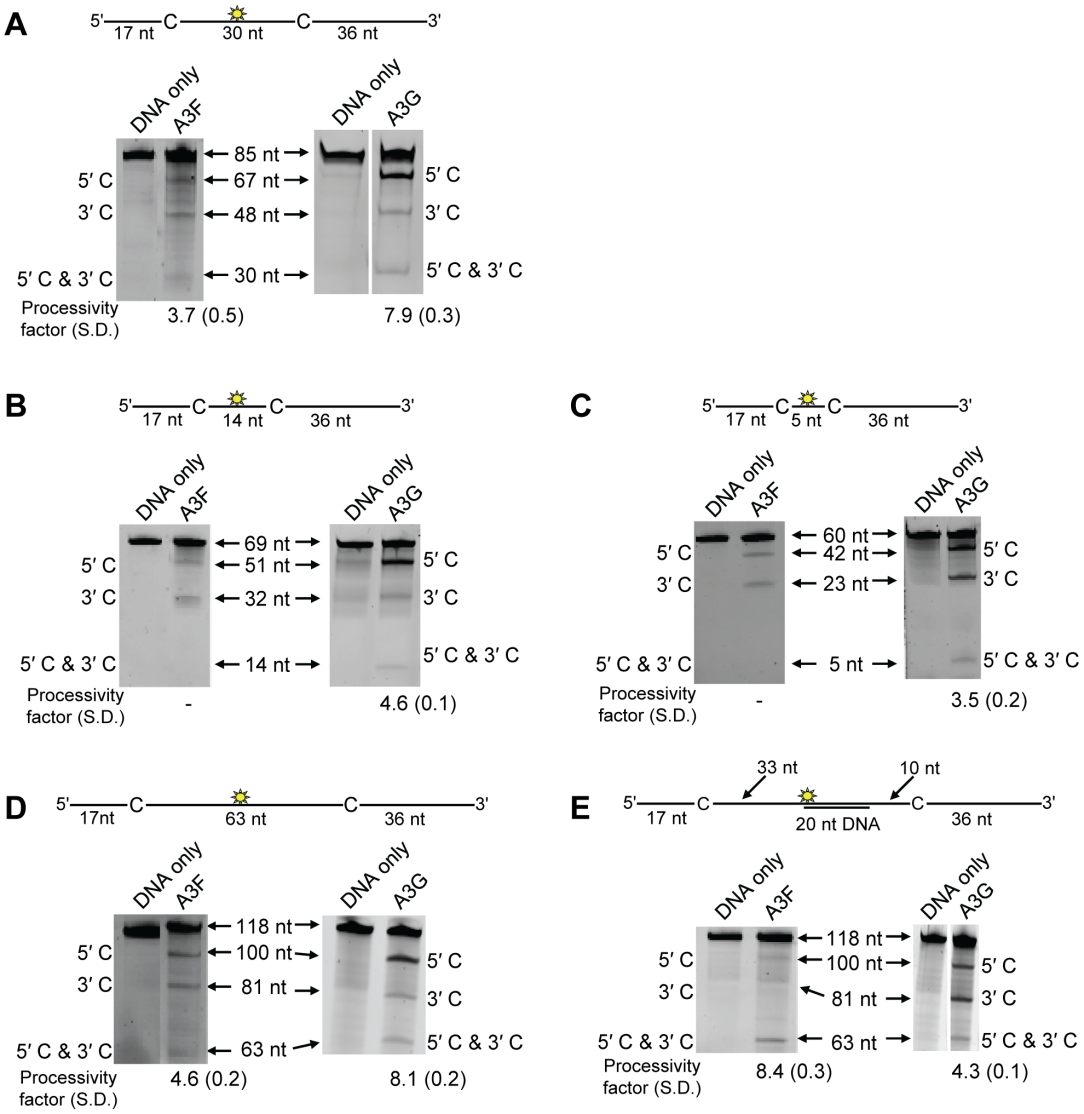
A3F and A3G are distinctively processive. Processivity of A3F and A3G were tested on substrates that contained an internal fluorescein (F)-label (yellow star) and two deamination motifs separated by different distances. The A3F substrates had 5′TTC motifs and the A3G substrates had 5′CCC motifs. (A) The two target cytosines within the 85 nt ssDNA sequence are spaced 30 nt apart. Single deaminations of the 5′C and 3′C are detected as the appearance of labeled 67- and 48- nt fragments, respectively; double deamination of both C residues on the same molecule results in a 30 nt labeled fragment (5′C & 3′C). (B) The two target cytosines within the 69 nt ssDNA sequence are spaced 14 nt apart. Single deaminations of the 5′C and 3′C are detected as the appearance of labeled 51- and 32- nt fragments, respectively; double deamination of both C residues on the same molecule results in a 14 nt labeled fragment (5′C & 3′C). (C) The two target cytosines within the 60 nt ssDNA sequence are spaced 5 nt apart. Single deaminations of the 5′C and 3′C are detected as the appearance of labeled 42- and 23- nt fragments, respectively; double deamination of both C residues on the same molecule results in a 5 nt labeled fragment (5′C & 3′C). (D) The two target cytosines within the 118 nt ssDNA sequence are spaced 63 nt apart. Single deaminations of the 5′C and 3′C are detected as the appearance of labeled 100- and 81- nt fragments, respectively; double deamination of both C residues on the same molecule results in a 63 nt labeled fragment (5′C & 3′C). (E) Deamination of the substrate described for (D), but with a 20 nt ssDNA annealed between the two target cytosines to block the sliding component of processivity. The measurements of processivity (Processivity factor) and the Standard Deviation of the mean (S.D.) are shown below the gel. The A3F: DNA ratio was 2∶1 except for panel (A) in which a 1∶1 ratio was used. The A3G: DNA ratio was (A–B) 1∶10, (C) 1∶2.5, (D–E) 1∶20. Enzyme: DNA ratios were varied due to different specific activities of the enzyme on a given DNA substrate. Values are an average from three independent experiments.

On a substrate with the target cytosines separated by 30 nt (Figure 1A, sketch), A3F was able to catalyze processive deaminations. The processivity factor is a ratio of the frequency of double deaminations on a single substrate to the predicted frequency of double deaminations of a nonprocessive enzyme (see Materials and Methods). Therefore, the processivity factor of 3.7 for A3F (Figure 1A) means that in a single enzyme-substrate encounter A3F was 3.7-fold more likely to catalyze a processive deamination than a nonprocessive deamination. On the cognate A3G substrate, A3G was 2-fold more likely than A3F to catalyze a processive deamination (compare Figure 1A, A3F, processivity factor of 3.7 and A3G, processivity factor of 7.9), suggesting that the processive mechanisms of A3F and A3G differ. In addition, we observed a difference in the ability of A3F and A3G to catalyze 5′-end biased deaminations. Where A3G has been found to prefer deaminations towards the 5′-end of ssDNA molecules due to a catalytic orientation specificity [35], A3F had a minimal 5′-end bias (Figure 1A, compare intensity of 5′C & 3′C bands for A3F and A3G). However, the presence or absence of a 5′-end bias does not influence the processivity calculation [47]. Since A3G has been found to use a dual sliding and jumping motion to scan ssDNA [34], [35], [37], we investigated whether the difference between A3F and A3G was due to a difference in the contributions of sliding and jumping or a different mode of scanning, e.g., intersegmental transfer.

First, we investigated the sliding ability of A3F. We conducted deamination assays on ssDNA substrates with closely spaced deamination targets, since it has been shown that sliding motions increase the frequency of closely spaced deaminations occurring processively [34]. With cytosines 14- and 5-nt apart, A3F was unable to catalyze any detectable processive deaminations (Figure 1B–C, A3F, absence of 5′C & 3′C band) indicating that A3F does not use sliding motions to catalyze processive deaminations. Of note, outside of single hit conditions (>15% substrate usage) we detected the band corresponding to deamination of both 5′TTC motifs on an ssDNA (5′C & 3′C band), which demonstrated that multiple molecules of A3F were able to deaminate these substrates at both cytosine targets to near completion (data not shown). In contrast, A3G was able to processively deaminate closely spaced residues under single-hit conditions by sliding (Figure 1B–C, A3G, processivity factors of 4.6 and 3.5).

Since A3F was processive on the substrate with the target cytosines separated by 30 nt (Figure 1A, A3F), but not on substrates with closely spaced deamination motifs (Figure 1B–C, A3F), the data suggested that A3F may use jumping or intersegmental transfer to processively deaminate cytosines. To investigate this further we determined the processivity of A3F on an ssDNA with the target cytosines separated by 63 nt (Figure 1D). On this substrate, A3F exhibited a processivity factor of 4.6 (Figure 1D, A3F), which is higher than the processivity factor obtained on the substrate with the target cytosines separated by 30 nt (Figure 1A, A3F, processivity factor of 3.7). In contrast, A3G which can slide and jump [35] maintained a processivity factor of ∼8 (compare Figure 1A and D, processivity factors). To confirm that we would observe only jumping or intersegmental transfer and not sliding motions, we annealed a 20 nt complementary DNA in between the target cytosines (Figure 1E, sketch). The double-stranded DNA portion is not bound as tightly by A3F (Figure S1A–B) or A3G (Figure S1C and [35], [37], [48], [49]) as ssDNA (Table 1) and results in the assay conditions blocking the sliding portion of the scanning activity [35], [47]. A3G was still processive on this substrate due to the ability to translocate on DNA in 3-dimensions by jumping, but we observed a ∼2-fold decrease in A3G processivity as compared to the analogous ssDNA substrate (Figure 1D–E, compare A3G processivity factors). We interpret that the ∼2-fold decrease in A3G processivity is due to A3G molecules attempting to slide over the dsDNA which induces dissociation from the DNA substrate and diffusion into the bulk solution. For A3F we observed a 1.8-fold increase in the processivity factor when we annealed a 20 nt complementary DNA in between the target cytosines (Figure 1D–E, compare A3F processivity factors), despite having a reduced binding to the double-stranded (ds)DNA portion (Figure S1A–B). The double deaminations became so efficient that the 5′- and 3′-proximal cytosine deaminations were barely visible on the gel (Figure 1E, A3F). A3F bound the 118 nt ssDNA substrate (Figure 1D) with an apparent K_d_ of 20 nM (Table 1), which is ∼7-fold lower than the apparent K_d_ of A3G (Table 1, K_d_ of 130 nM). This indicates that A3F is less likely to dissociate from an ssDNA substrate than A3G, but does not fully explain why we observed an increase in processivity by annealing a complementary DNA in between the target cytosines. Results were not changed by annealing a 20 nt complementary RNA molecule to the substrate (Figure S2A–B) or by testing A3F on a different partially dsDNA substrate which contained two 5′ATC motifs (Figure S3). We speculated that the processivity of A3F increased as opposed to remaining the same in the presence of the complementary DNA because the structural change in the substrate induced by the dsDNA region made jumping events more successful. This could occur if the average jumping distance of A3F were different than A3G and the rigid dsDNA region juxtaposed the 5′TTC motifs at a distance which was highly accessible by A3F.

**Table 1:** Comparison of apparent dissociation constants (K_d_) from ssDNA of A3G and A3F wild-type and mutants.

Enzyme	K_d_ (nM)
A3G	130±6
A3F	20±1
A3F CTD	288±10
A3G NPM	56±4
A3F NGM	119±11

**Figure 2:**
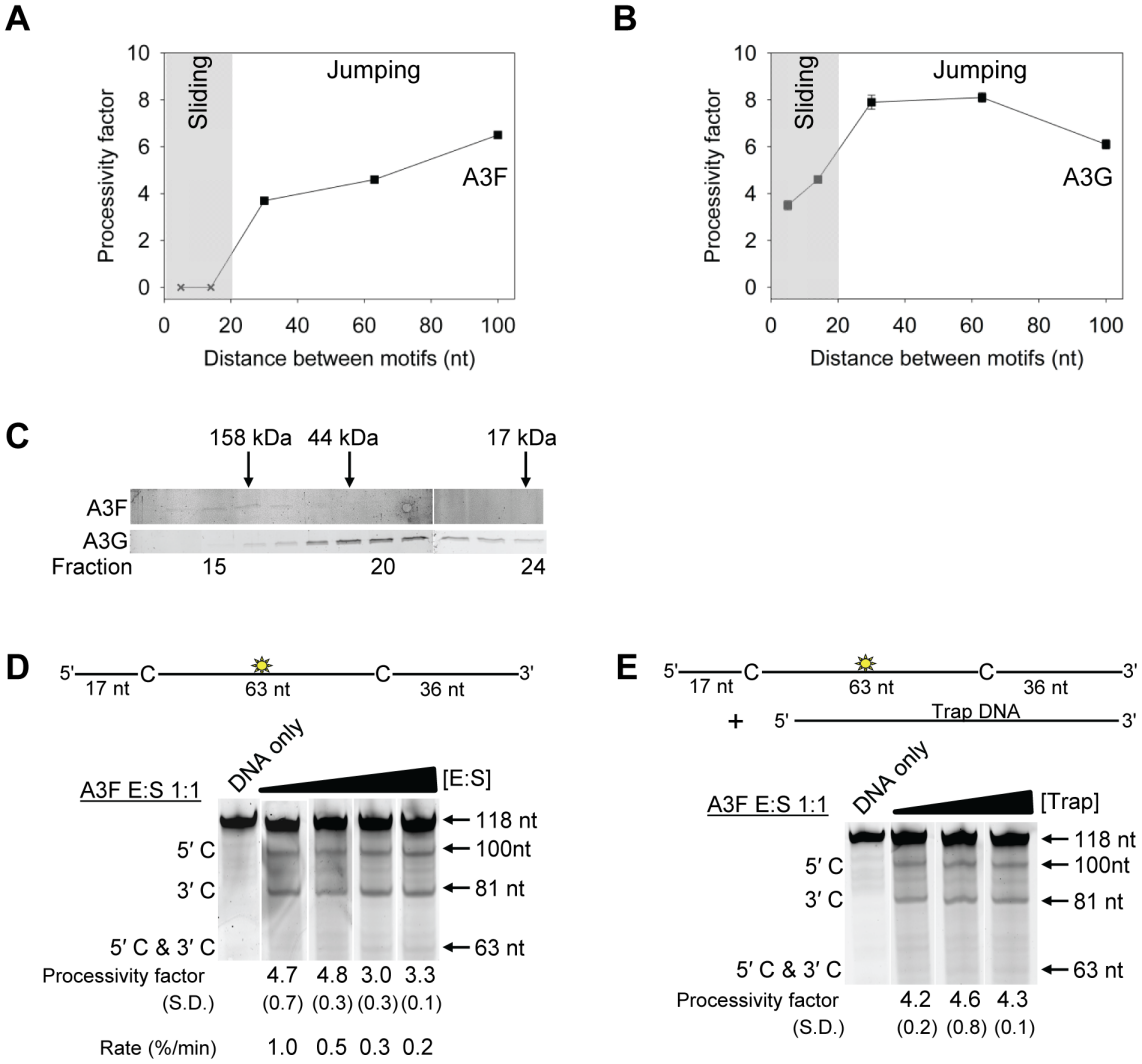
A3F translocations on ssDNA are distinct from A3G, but not due to intersegmental transfer. (A–B) Summary of processivity factors for (A) A3F and (B) A3G on ssDNA substrates where the two deamination motifs were separated by 5- to 100- nt. A3F processivity was not observable until the distance between cytosines was greater than 14 nt apart (denoted with x), but then increased until 100 nt (filled squares). This was distinct from A3G that was processive when cytosines were closely spaced (5- to 14- nt apart), reached a maximum processivity when cytosines were 30- to 63- nt apart and then decreased in processivity (filled squares). The grey area represents the region where sliding is required for processivity. Jumping has been previously defined to be translocations of ≥20 nt [72]. Gels for the substrate with deamination motifs separated by 100 nt are shown in Figure S4. (C) Size exclusion chromatography demonstrates that A3F forms tetramers (∼180 kDa) and higher order oligomers. This is in contrast to A3G which forms monomers (∼46 kDa) and dimers. (D–E) Processivity of A3F was tested on a substrate that contained an internal fluorescein (F)-label (yellow star) and two deamination motifs (5′TTC) separated by 63 nt. Single deaminations of the 5′C and 3′C are detected as the appearance of labeled 100- and 81- nt fragments, respectively; double deamination of both C residues on the same molecule results in a 63 nt labeled fragment (5′C & 3′C). (D) The enzyme: substrate ratio of 1∶1 (E∶S) was kept constant, but reaction components increased (100∶100, 200∶200, 300∶300, 400∶400 nM) to investigate whether A3F could transfer between two ssDNA substrates. (E) In the presence of an unlabeled ssDNA trap (69 nt) the processivity factor of A3F (E∶S of 1∶1) remained the same regardless of trap concentration (1∶0.5, 1∶1, or 1∶5 ratio of labeled ssDNA to unlabeled trap ssDNA). The measurements of processivity (Processivity factor), Standard Deviation of the mean (S.D.), and Rate (%/min) are shown below the gels. Values are an average from at least two independent experiments.

To test this hypothesis we examined the processive deaminations of A3F and A3G on an ssDNA substrate with deamination motifs separated by 100 nt. We found that as the distance between deamination motifs was increased up to 100 nt, the processivity factors of A3F also increased (Figure 2A). In contrast, A3G processivity exhibited a plateau when deamination motifs were 30- to 63-nt apart and the processivity factor decreased when deamination motifs were 100 nt apart (Figure 2B). These data demonstrate that the average jumping distance of A3F and A3G differ. Similar results were also found from analysis of deamination-induced mutations in the model HIV replication assay and are discussed later in the text. To identify a possible reason for the different jumping ability of A3F we examined its oligomerization state in comparison to A3G. A3G is known to form polydisperse oligomers that are dependent on enzyme concentration and buffer conditions [50]. Using size exclusion chromatography at low enzyme concentrations we found that A3F formed predominantly tetramers (∼180 kDa) and higher order oligomers whereas A3G eluted as predominantly a monomer (∼46 kDa) with minor dimeric species (Figure 2C). The finding that A3F forms more tetramers than A3G is consistent with previous sucrose gradient data [51] and data on the CTD portions of these enzymes. The A3F CTD can oligomerize more readily than the A3G CTD [52], [53], [54], [55]. The A3F oligomers remained soluble as high speed centrifugation did not result in a discernable protein pellet. These data demonstrated that A3F oligomers are more stable than A3G oligomers at low protein concentration and suggest a structural difference that could account for why the A3F jumping distance is different than A3G (Figure 2A–B).

However, since the A3F ssDNA scanning mechanism is more efficient in distal translocations (Figure 1), we also investigated whether it was scanning ssDNA by intersegmental transfers, rather than or in addition to jumping. This mode of DNA scanning involves an enzyme molecule that binds in two distal locations on the DNA before completing the translocation by dissociating from one location [39], [40]. The intersegmental transfer mechanism requires that the enzyme have more than one DNA binding domain. A3F could bind ssDNA with both its NTD and CTD on one or many subunits of the oligomer. This is in contrast to jumping which uses microdissociations and reassociations to scan ssDNA [38], [41]. A key difference between jumping and intersegmental transfer is that the probability of an enzyme transferring to another DNA substrate is low for jumping but high for intersegmental transfer [39], [40], [56]. Therefore, to observe whether A3F can scan ssDNA by intersegmental transfer we increased the enzyme and substrate concentrations, but kept their ratio constant. Crowding the reaction in this manner with enzyme and ssDNA can increase the tendency of the enzyme to translocate to a different ssDNA if intersegmental transfer is occurring [56]. This would result in a decrease in the observed processivity with increasing reaction components. We found that A3F maintained the same processivity at a 1∶1 E∶S ratio at concentrations of 100 nM and 200 nM (Figure 2D, processivity factor of 4.7 and 4.8). At a 1∶1 E∶S ratio using concentrations of 300 nM and 400 nM the processivity of A3F decreased ∼1.5-fold from 4.7 to 3.0 or 3.3 (Figure 2D), providing evidence that A3F can use intersegmental transfer to scan ssDNA. However, the decrease in A3F processivity is small (∼1.5 fold), does not decrease gradually with increasing enzyme and substrate concentration, and is not completely abolished (processivity factor remains above 1) suggesting that intersegmental transfer is not the primary mechanism of DNA scanning, but can occur in a minority of ssDNA-A3F interactions. Importantly, intersegmental transfer should result in an increase in the reaction rate with increasing DNA concentration since the rate of searching is enhanced by increasing the apparent off rate, which allows more rapid sampling of DNA [56]. However, the reaction rate of A3F decreased with increasing enzyme and substrate concentrations (Figure 2D, Rate) and supports the conclusion that intersegmental transfer is not a primary mode of scanning ssDNA. In further support of this interpretation is that we only observed evidence of intersegmental transfer with increasing enzyme and substrate concentration (Figure 2D), not when the ssDNA concentration alone was increased (Figure 2E, processivity factors of 4.2 to 4.6), which indicates that A3F does not readily transfer to another ssDNA without high local concentrations of enzyme, i.e., the intersegmental transfer is not inherent to A3F but requires excessive crowding of reaction conditions. A3G showed no decrease in processivity with increasing concentration of enzyme and substrate, despite also containing both a NTD and CTD (Figure S5). This difference may arise since the CTD of A3G binds ssDNA in the micromolar range [54], [55], [57], in contrast to the CTD of A3F that can bind DNA in the nanomolar range (Table 1, apparent K_d_ of 288 nM). All together the data supported the conclusion that A3F primarily utilized jumping and not intersegmental transfer to scan ssDNA.

### The A3F DNA scanning mechanism does not enable efficient mutagenesis of (−)DNA

Our biochemical data on synthetic substrates (Figure 1) predicts that A3F will not efficiently catalyze deaminations during proviral DNA synthesis due to a predominant jumping movement that would result in a superficial scan of the ssDNA [34], [43]. Importantly, we observed this predominant jumping movement when A3F encountered an RNA/DNA hybrid (Figure S2A), such as would be encountered during synthesis of the HIV provirus. To test this prediction we used our model *in vitro* HIV replication system. Since this system reconstitutes reverse transcription of (−)DNA and synthesis of (+)DNA, it allows us to observe the ability of A3 enzymes to induce mutagenesis in a dynamic system, such as occurs *in vivo*, but with the advantage of controlling the amount of enzyme added to the reaction system. Specifically, this system uses an *in vitro* synthesized RNA which contains (from the 5′-end) a polypurine tract (PPT), part of the protease gene (*prot*) of HIV, and a *lacZα* reporter. The RNA is reverse transcribed to (−)DNA by reverse transcriptase and after RNaseH-mediated removal of the RNA, the PPT enables (+)DNA synthesis without the addition of an exogenous primer. In this manner we can achieve the salient properties of HIV replication that A3 enzymes must contend with, a finite time to access single-stranded (−)DNA and a heterogeneous substrate that is interspersed with RNA fragments.

**Figure 3:**
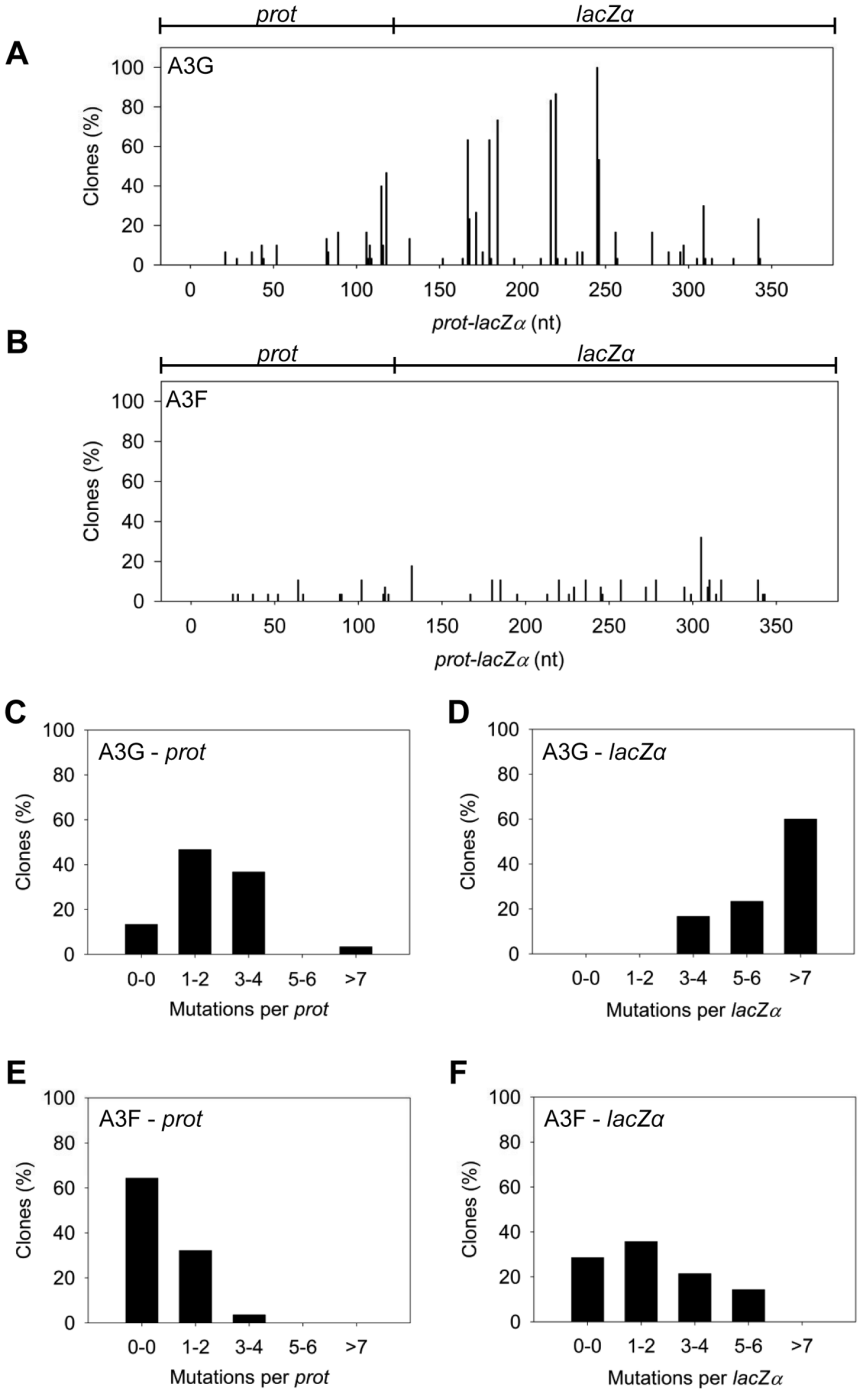
A3F exhibits low mutagenic potential in a model HIV replication system. (A–B) Spectra of mutations are plotted as the percentage of clones containing a mutation at a particular location (nt) in the 368 nt *prot-lacZ*α construct for (A) A3G or (B) A3F. (C–F) Histograms illustrate the disparity between the number of mutations that can be induced by A3G versus A3F in the (C, E) *prot* region that is single stranded for a shorter time than the (D, F) *lacZα* region.

The A3G data demonstrated the potential amount of mutations that could occur in this system. A3G had a clonal mutation frequency of 2.63×10^−2^ mutations/bp which is 10-fold over the background mutation frequency of reverse transcriptase (RT) (Table 2). Further, the A3G mutation spectra have clear hot-spots at 5′CCC or 5′CC motifs in both the *prot* and *lacZα* with some sites being mutated in 100% of clones (Figure 3A, e.g., 245 nt). Due to the PPT being nearest the *prot*, this region is converted to dsDNA the fastest and incurs less mutations than regions nearer the center or 3′-end of the (+)DNA (Figure 3A). As such, we can recover white colonies indicating a mutation in the *lacZα* reporter but upon sequencing find no mutations in the *prot*. Therefore, the number of clones with mutations in the *prot* is a measure of how efficiently an A3 enzyme can induce mutations. The *lacZα* remains single stranded longer and can therefore be visited by multiple A3 enzymes multiple times. In the *prot* region, A3G was found to induce no mutations in 13% of clones, but the majority of clones had either 1–2 mutations (47%) or 3–4 mutations (37%) (Figure 3C). In the *lacZα*, A3G-induced mutagenesis resulted in >7 mutations in the majority of clones (Figure 3D, 60%).

**Table 2:** A3-mediated mutation frequencies in a model HIV replication system.

Enzyme	Population mutation frequency	Base pairs sequenced	Total number of G→A mutations	Clone mutation frequency (×10^−2^ mutations/bp)
A3G	0.89	11 040	290	2.63
A3F	0.61^a^	10 304	78	0.76^a^
A3G NPM	0.14^a^	9200	27	0.29^a^
A3F NGM	0.80	10 672	36	0.34^a^

The ratio of white colonies to total colonies is defined as the population mutation frequency. The average number of G→A mutations per base pair in the 368 nt *prot-lacZα* construct is defined as the clone mutation frequency. For RT alone (no A3), all base changes were used to calculate the clone random mutation frequency which established a baseline of 0.27×10^−2^ mutations/bp. The RT alone condition produced a population mutation frequency of 0.07.

a Significant difference was p≤0.001 versus A3G values.

Addition of A3F to the model HIV replication assay resulted in a modest 2.8-fold increase over the background mutation frequency (Table 2). Examination of the mutation spectrum demonstrated that A3F could induce mutagenesis at a number of 5′TTC or 5′TC sites along the *prot* and *lacZα*, but that there were no clear hot-spots, except possibly at position 305 nt of *lacZα* (Figure 3B). This may be due to the random binding of A3F to the (−)DNA and an inefficient search of the enzyme by jumping without local scanning by sliding (Figure 1), which would make interaction with multiple 5′TTC or 5′TC motifs less likely to occur. Of note, the mutation frequencies induced by A3F and A3G did not increase with the addition of more enzyme to the reaction demonstrating that both A3F and A3G are present at saturating levels (data not shown). Analysis of the distances between A3F-induced mutations demonstrated that 75% of the mutations were separated by more than 20 nt (Table 3), confirming that A3F was using jumping this assay system, in agreement with the data on the synthetic oligonucleotide substrates (Figure 1). In contrast, only 50% of A3G-induced mutations were separated by more than 20 nt (Table 3), providing confirmation that A3G is capable of recognizing sites that are more closely spaced (Figure 2A–B). The analysis in Table 3 included all clones (highly mutated and sparsely mutated). To ensure we did not bias our analysis we also examined only sparsely mutated clones for both A3G and A3F (2–5 mutations) and obtained similar results for frequency of mutations separated by more than 20 nt (A3G, 60% and A3F, 85%). In addition, we hypothesized that the tight binding of A3F to ssDNA (Table 1), would prevent A3F from frequently dissociating into the bulk solution and reassociating with different (−)DNAs. In agreement with the binding data, A3F increased the population mutation frequency (frequency of white colonies) only 9-fold over the background whereas A3G caused a 12-fold increase in the population mutation frequency (Table 2). Although the overall level of mutagenesis induced by A3F was low, we did observe slightly more mutations in the *lacZα* than the *prot* region due to the replication kinetics (Figure 3E–F). In the majority of A3F clones (64%) there were no mutations in the *prot* region (Figure 3E). In the *lacZα* region the majority of clones only had 1–2 mutations (Figure 3F, 36%). However, 29% of clones did not have a G→A mutation and were recovered due to an RT induced error (Figure 3F, 0-0). These data demonstrated that A3F was inefficient at inducing mutagenesis during reverse transcription even in areas where the enzyme had ample time to access ssDNA (*lacZα*) and especially in regions that are single-stranded the shortest time (*prot*) (Figure 3E–F).

**Table 3:** Analysis of distances between G→A mutations for A3G and A3F.

Enzyme	Frequency of clones with mutations >20 nt apart
A3G	0.51
A3F	0.75^a^

a Significant difference was p≤0.001 versus A3G values.

### Inactivation of HIV *prot*

**Figure 4:**
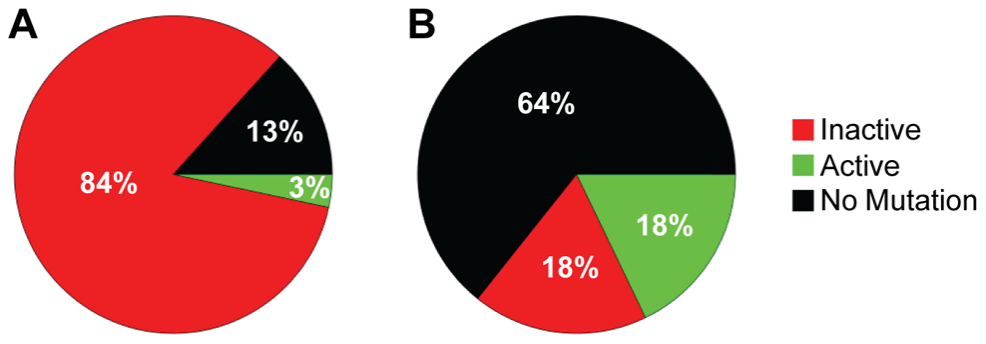
Predicted ability of A3F and A3G to inactivate HIV protease. Each *prot* clone was individually analyzed to determine the percentage of clones that resulted in a mutated and inactive (red) *prot*, mutated and active *prot* (green) or *prot* with no mutations (black) after exposure to (A) A3G or (B) A3F. (A) A3G was able to inactivate the *prot* in 84% of clones and left an active *prot* in 3% of mutated clones. A3G did not induce any mutations in the *prot* in 13% of clones. (B) A3F-induced mutagenesis was less effective than A3G due to no mutations being induced in 64% of clones. Of the 36% of clones with a mutation, 18% left the *prot* active and 18% inactivated the *prot*.

The increased distance between A3F-induced mutation sites in the HIV replication assay (Table 3) in combination with the data on synthetic oligonucleotide DNA indicating that A3F prefers to use jumping (Figure 1) provides evidence that the decreased mutagenic ability observed for A3F in cell culture may be due to an inefficient search mechanism on DNA. However, these observations are inconsequential if each mutation by A3F were to inactivate the *prot* gene, which is used here as a predictor of HIV inactivation potential. We gauged the probability that the *prot* of HIV would be inactivated by A3F by determining the mutated amino acid sequences and comparing this to an extensive mutagenesis study of the *prot* conducted by Loeb *et al.* [58]. Consistent with A3F inducing a low number of mutations (Figure 3 and Table 2), there were no A3F-induced mutations in 64% of clones (Figure 4B). On a per clone basis, A3F-induced mutations resulted in protease inactivation only 50% of the time (Figure 4B, 18% active and 18% inactive). The high number of clones remaining active was due to two reasons. First, some clones incurred mutations in regions where any amino acid is tolerated [58], even nonconservative changes, e.g., E21K, so the mutation was insignificant (Table S2). Second, some clones incurred a mutation that resulted in a conservative change to the amino acid which enabled the protease to retain full or partial activity, depending on the proximity to the active site residues [58] (Table S2). For example, the M46I mutation was induced by A3F in 11% of clones, but results an active and drug resistant protease. The remainder of the A3F-induced mutations were found only in single clones and 36% of those mutations resulted in an active protease (Table S2). All together, A3F was not efficient at inactivating the HIV protease and could also induce resistance to protease inhibitors (Table S2, D30N and M46I). This was in contrast to A3G which caused inactivation of 84% of the clones and left only 3% of clones active (Figure 4A). A3G also did not mutate some *prot* clones, but only 13% of the time (Figure 4A). A3G did induce protease drug resistant mutations in 10% of the population (Table S2, D30N), but the examination of these clones individually demonstrated that they were inactivated by other mutations. Overall, we found that per mutation A3G was more likely to cause an inactivating mutation than A3F. This appeared to be due to the ability of a 5′CCC motif to cause more nonconservative mutations than 5′TTC in the *prot* (Table S2). For example, A3G had clear hot spots that caused inactivation of the protease, e.g., W42 STOP, 20% of clones; G51R, 36% of clones; G52S, 52% of clones (Table S2).

### Determinants of processivity for A3 enzymes

**Figure 5:**
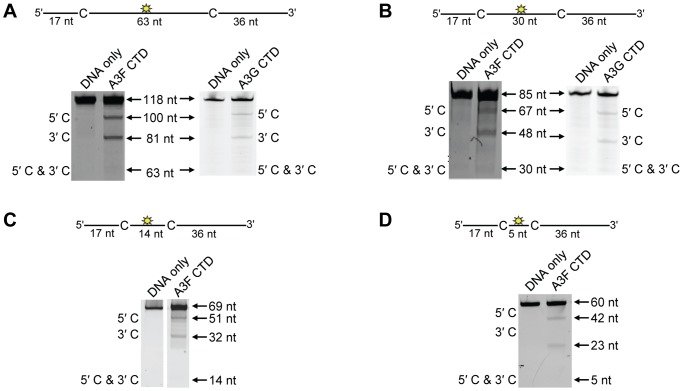
Similar to the A3G CTD, the A3F CTD does not deaminate cytosines processively. Processivity of A3F CTD and A3G CTD were tested on substrates that contained an internal fluorescein (F)-label (yellow star) and two deamination motifs separated by different distances. The substrates had 5′TTC motifs (A3F CTD) or 5′CCC motifs (A3G CTD). (A) The two target cytosines within the 118 nt ssDNA sequence are spaced 63 nt apart. Single deaminations of the 5′C and 3′C are detected as the appearance of labeled 100- and 81- nt fragments, respectively. Double deamination of both C residues on the same molecule, which would result in a 63 nt labeled fragment (5′C & 3′C), could not be detected indicating that A3F CTD and A3G CTD are not processive on these ssDNA substrates. (B) The two target cytosines within the 85 nt ssDNA sequence are spaced 30 nt apart. Single deaminations of the 5′C and 3′C are detected as the appearance of labeled 67- and 48- nt fragments, respectively. Double deamination of both C residues on the same molecule, which would result in a 30 nt labeled fragment (5′C & 3′C), could not be detected indicating that A3F CTD and A3G CTD are not processive on these ssDNA substrates. (C) The two target cytosines within the 69 nt ssDNA sequence are spaced 14 nt apart. Single deaminations of the 5′C and 3′C are detected as the appearance of labeled 51- and 32- nt fragments, respectively. Double deamination of both C residues on the same molecule, which would result in a 14 nt labeled fragment (5′C & 3′C), could not be detected indicating that A3F CTD is not processive on this ssDNA substrate. (D) The two target cytosines within the 60 nt ssDNA sequence are spaced 5 nt apart. Single deaminations of the 5′C and 3′C are detected as the appearance of labeled 42- and 23- nt fragments, respectively. Double deamination of both C residues on the same molecule, which would result in a 5 nt labeled fragment (5′C & 3′C), could not be detected indicating that A3F CTD is not processive on this ssDNA substrate. The A3F CTD: DNA and A3G CTD: DNA ratios were 2∶1. A representative gel from three independent experiments is shown.

To investigate the A3F DNA scanning mechanism further we made mutants in A3G and A3F to alter their processive scanning behavior. For A3G, the only other A3 double Z-domain enzyme studied with regards to processivity, the NTD domain acts as a processivity factor [34]. The A3G CTD domain alone is non-processive (Figure 5A–B and [53], [54]). In order to focus in on the determinants of processivity, we recombinantly expressed the CTD domain of A3F and tested its processivity using ssDNA substrates as in Figure 1. We found that the CTD of A3F could not processively deaminate cytosines that were spaced 63- or 30- nt apart, similar to the CTD of A3G (Figure 5A–B, absence of 5′C & 3′C band). The A3F CTD could also not processively deaminate target cytosines 14- or 5-nt apart (Figure 5C–D, absence of 5′C & 3′C band), similar to the full-length A3F enzyme (Figure 1B–C). These data indicated that the NTD of A3F was a processivity factor.

**Figure 6:**
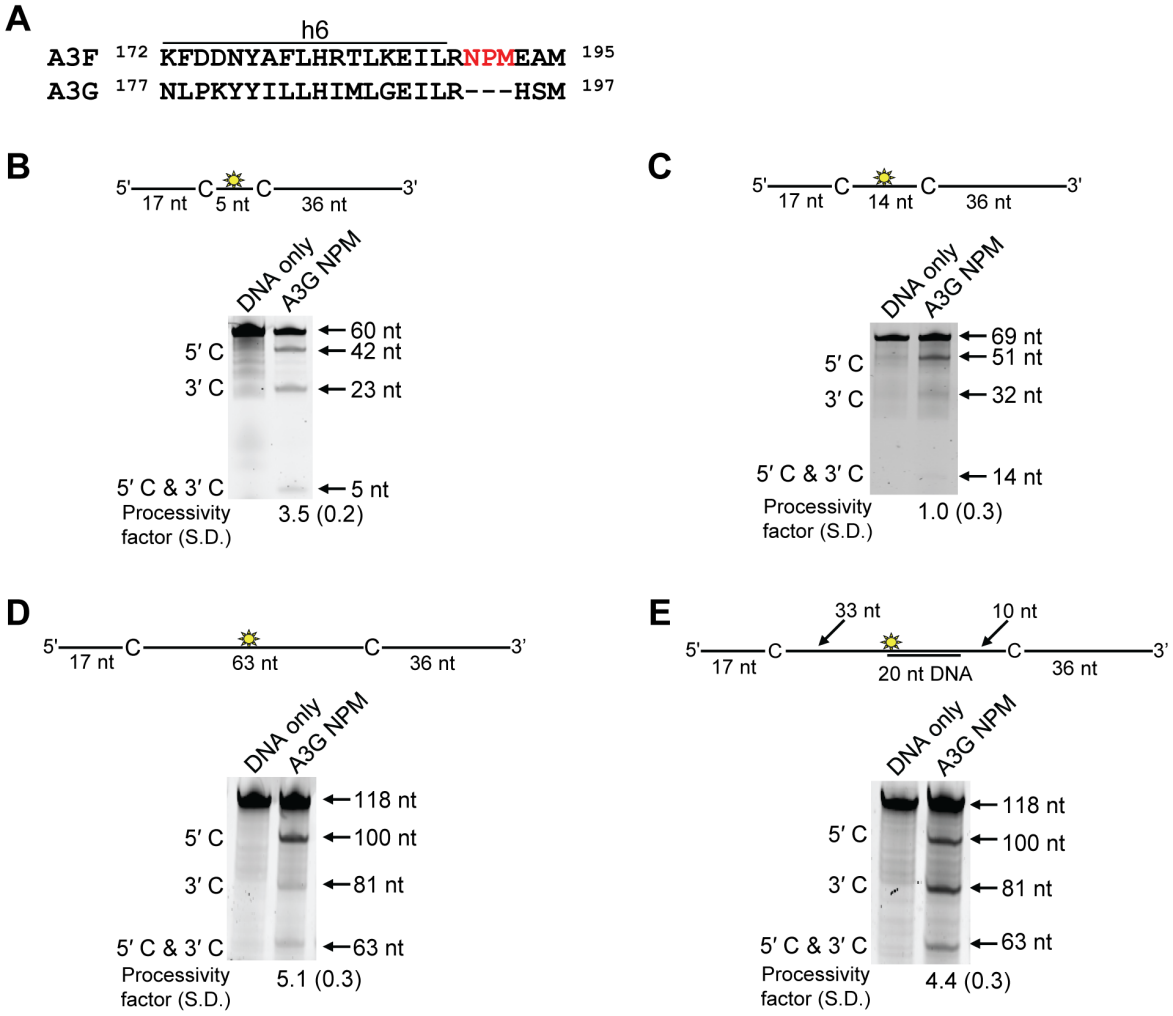
Decreased processivity of A3G NPM was attributable to changes in sliding, but not jumping movements. (A) The A3G NPM mutant was created by inserting the NPM motif found in A3F into A3G immediately after Arg 194. (B–E) Processivity of A3G NPM was tested on substrates that contained an internal fluorescein (F)-label (yellow star) and two deamination motifs separated by different distances. The substrates had 5′CCC motifs. (A) The two target cytosines within the 60 nt ssDNA sequence are spaced 5 nt apart. Single deaminations of the 5′C and 3′C are detected as the appearance of labeled 42- and 23- nt fragments, respectively; double deamination of both C residues on the same molecule results in a 5 nt labeled fragment (5′C & 3′C). (C) The two target cytosines within the 69 nt ssDNA sequence are spaced 14 nt apart. Single deaminations of the 5′C and 3′C are detected as the appearance of labeled 51- and 32- nt fragments, respectively. Double deamination of both C residues on the same molecule results in a 14 nt labeled fragment (5′C & 3′C) and were detected at a low level resulting in a processivity factor of 1 (below gel). Since the processivity factor is a ratio between the observed double deaminations and the theoretical deaminations expected to occur for a nonprocessive enzyme (see Materials and Methods), the results indicated that the A3G NPM mutant was not processive on this substrate. (D) The two target cytosines within the 118 nt ssDNA sequence are spaced 63 nt apart. Single deaminations of the 5′C and 3′C are detected as the appearance of labeled 100- and 81- nt fragments, respectively; double deamination of both C residues on the same molecule results in a 63 nt labeled fragment (5′C & 3′C). (E) Deamination of the substrate described for (D), but with a 20 nt ssDNA annealed between the two target cytosines to block the sliding component of processivity. The measurements of processivity (Processivity factor) and the Standard Deviation of the mean (S.D.) are shown below the gel. The A3G NPM: DNA ratio was (B) 1∶2.5, (C) 1∶10, (D–E) 1∶20. Enzyme: DNA ratios were varied due to different specific activities of the enzyme on a given DNA substrate. Values are an average from three independent experiments.

To determine the specific amino acids within the NTD that differentiate the processive scanning behaviors of A3F and A3G we aligned their amino acid sequences and looked for differences in the predicted helix 6 and loop 7 (Figure S6) since these regions have been shown to influence the scanning behavior of A3G [34]. Specifically, it was found that helix 6 mediated sliding movements and loop 7 mediated jumping movements [34]. Since we could not observe any scanning by sliding for A3F (Figure 1B–C), we hypothesized that residues within or near predicted N-terminal helix 6 would be different from A3G. For A3G, His186 was found to be essential for sliding movements [34]. Although A3F has a His181 equivalent to A3G (His186), at the end of the predicted helix 6 in the connection domain between the NTD and CTD, A3F has an additional three amino acids, ^190^NPM^192^, in comparison to A3G (Figure 6A). To test whether the ^190^NPM^192^ motif prevents A3F from sliding, we inserted the NPM motif into the equivalent position in A3G (^195^NPM^197^) creating an A3G NPM mutant. We then tested if A3G NPM was still able to undergo scanning by sliding. Using the ssDNA substrates with target cytosines close together enables the observation of processive deaminations by sliding [34]. On the substrate with cytosines separated by 5 nt, A3G NPM retained its processivity at an equivalent frequency to that of the wild-type A3G (compare Figure 6B and Figure 1C, processivity factors). On the substrate with cytosines separated by 14 nt, A3G NPM was essentially not processive, as evidenced by a processivity factor of 1 which means that A3G NPM double deaminations occurred at the same frequency as expected if they were uncorrelated (Figure 6C). This was in contrast to wild-type A3G that was able to processively deaminate cytosines located 14 nt apart (Figure 1B, processivity factor 4.6). These data indicated that the NPM insertion had decreased the sliding distance of A3G. To ensure that jumping was not affected, we tested the A3G NPM mutant on a substrate with cytosines separated by 63 nt without or with a complementary DNA or RNA annealed. First, we established the processivity on this substrate when fully single-stranded. Accordingly, the A3G NPM which had a decreased ability to slide, had a decreased processivity factor on this substrate in comparison to wild-type A3G (compare Figure 6D, processivity factor of 5.1 to Figure 1D, processivity factor of 8.1), but similar to A3F (Figure 1D, processivity factor of 4.6). When a complementary DNA was annealed the processivity of the A3G NPM was not decreased (Figure 6E, processivity factor of 4.4) demonstrating that the jumping motion of A3G NPM was not affected. Similar results were found when a complementary RNA was annealed to the substrate (Figure S2C). This was in contrast to the characteristic 2-fold decrease in processivity observed with A3G when a complementary DNA or RNA is annealed in between two target cytosines (Figure 1D–E and Figure S2B) consistent with the hypothesis that attempts to slide over the dsDNA region by wild-type A3G results in dissociation of the enzyme into the bulk solution. That we did not find an increase in the jumping efficiency for the A3G NPM (Figure 6E), in contrast to A3F (Figure 1E) is in agreement with published data that suggest the determinants of jumping are separate from sliding and localized to predicted loop 7 (Figure S6 and [34]). Further, the oligomerization state of A3G NPM is equivalent to wild-type A3G (data not shown), not A3F and this may influence the jumping distance of an A3 enzyme (Figure 2A–C).

To ensure that the effects on processivity were due to specific changes to the residues interacting with ssDNA while scanning, rather than solely due to a poor affinity for ssDNA, we examined A3G NPM by circular dichroism (CD) spectroscopy and the ability of A3G NPM to bind ssDNA using rotational anisotropy. The CD analysis confirmed that A3G NPM and A3G were structurally similar (data not shown). Interestingly, addition of the NPM residues to A3G resulted in a 2-fold increase in the binding affinity of A3G for the ssDNA, implicating these residues in the ssDNA-NTD interaction (Table 1, A3G, K_d_ of 130 nM; A3G NPM, K_d_ of 56 nM). The specific activity of A3G NPM was decreased ∼3-fold in comparison to A3G (Table 4, A3G, 15 pmol/µg/min; A3G NPM, 5.5 pmol/µg/min).

**Table 4:** Specific activities of A3G and A3F wild-type and mutants.

Enzyme	Specific Activity (pmol/µg/min)
A3G	15±1
A3F	0.14±0.03
A3G NPM	5.5±1
A3F NGM	0.20±0.02

The specific activity was determined using the substrate with target cytosines separated by 63 nt (118 nt substrate) and the values are shown with the standard deviation that was calculated from three independent experiments.

**Figure 7:**
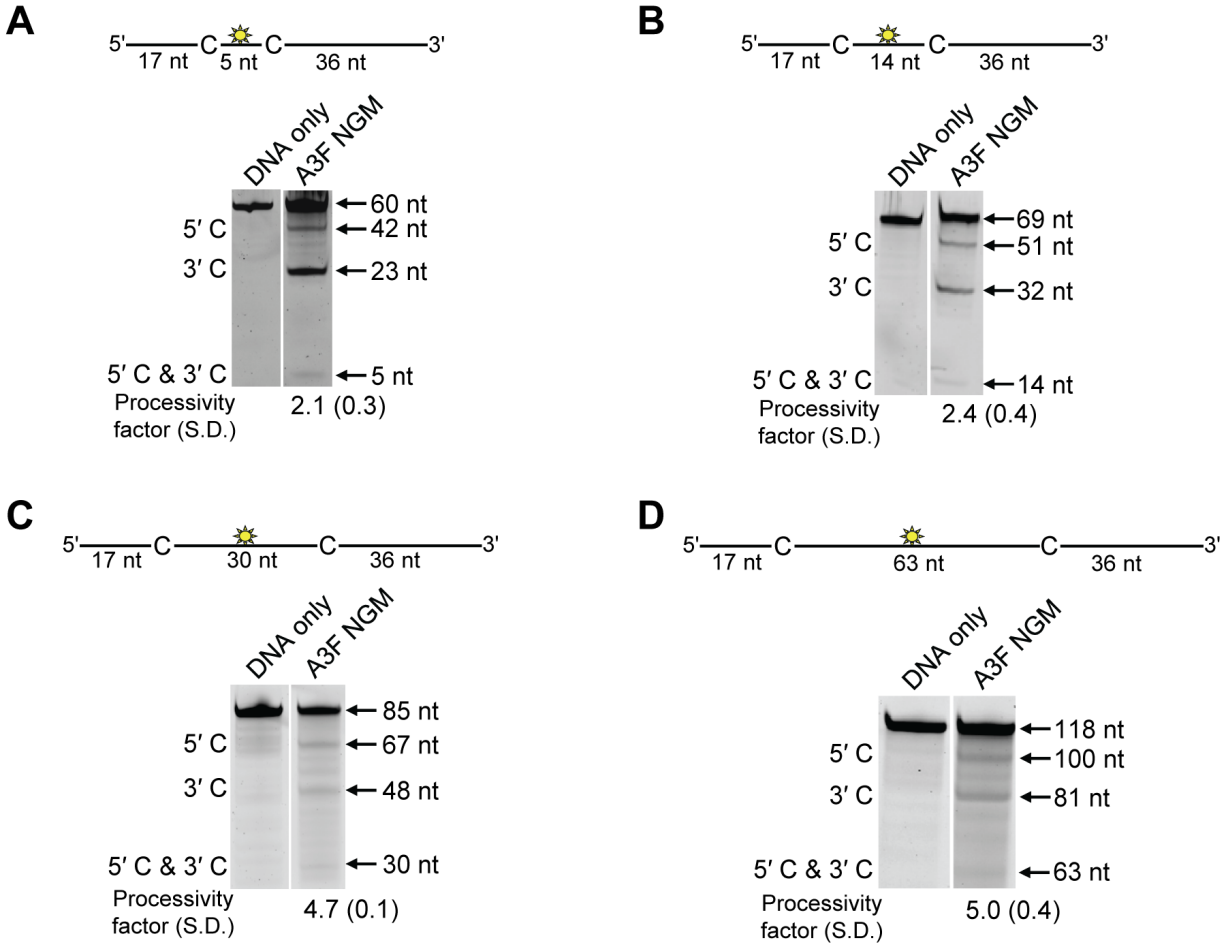
A3F NGM is able to slide on ssDNA to catalyze processive deaminations. Processivity of A3F NGM was tested on substrates that contained an internal fluorescein (F)-label (yellow star) and two deamination motifs separated by different distances. The substrates had 5′TTC motifs. (A) The two target cytosines within the 60 nt ssDNA sequence are spaced 5 nt apart. Single deaminations of the 5′C and 3′C are detected as the appearance of labeled 42- and 23- nt fragments, respectively; double deamination of both C residues on the same molecule results in a 5 nt labeled fragment (5′C & 3′C). (B) The two target cytosines within the 69 nt ssDNA sequence are spaced 14 nt apart. Single deaminations of the 5′C and 3′C are detected as the appearance of labeled 51- and 32- nt fragments, respectively. Double deamination of both C residues on the same molecule results in a 14 nt labeled fragment (5′C & 3′C). (C) The two target cytosines within the 85 nt ssDNA sequence are spaced 30 nt apart. Single deaminations of the 5′C and 3′C are detected as the appearance of labeled 67- and 48- nt fragments, respectively; double deamination of both C residues on the same molecule results in a 30 nt labeled fragment (5′C & 3′C). (D) The two target cytosines within the 118 nt ssDNA sequence are spaced 63 nt apart. Single deaminations of the 5′C and 3′C are detected as the appearance of labeled 100- and 81- nt fragments, respectively; double deamination of both C residues on the same molecule results in a 63 nt labeled fragment (5′C & 3′C). The measurements of processivity (Processivity factor) and the Standard Deviation of the mean (S.D.) are shown below the gel. The A3F NGM: DNA ratio was 1∶1. Values are an average from three independent experiments.

To further investigate the influence of the NPM motif in A3F, we attempted a reciprocal mutation, i.e., deleting the NPM motif from A3F. However, the mutant A3F did not express well in the *Sf*9 expression system indicating that the NPM deletion caused a structural instability. To circumvent this we made a conservative mutation in A3F to change the NPM motif to an NGM motif. We hypothesized that the Pro would have a significant influence on the functionality of the motif since Pro gives structural rigidity. We then tested the ability of the A3F NGM to processively deaminate two closely spaced deamination motifs by sliding. We found that A3F NGM was able to processively deaminate cytosines that were 5 nt and 14 nt apart (Figure 7A–B, processivity factors of 2.1 and 2.4), in contrast to A3F (Figure 1B–C). When the distance between the cytosines was increased to 30 nt or 60 nt apart, A3F NGM was able to undergo processive deaminations similarly to A3F (compare Figure 7C–D to Figure 1A and D). Interestingly, the apparent K_d_ of A3F NGM was 119 nM, which is 6-fold larger than the K_d_ of A3F (Table 1, 20 nM) further implicating these residues in the enzyme-ssDNA interaction. The specific activity of A3F NGM was ∼1.5-fold higher than A3F (Table 4). The A3F NGM and A3G NPM results demonstrated that the presence of an NPM motif blocks the ability of both A3F and A3G to processively slide on ssDNA.

### Contributions of processivity to efficient mutagenesis of (−)DNA

**Figure 8:**
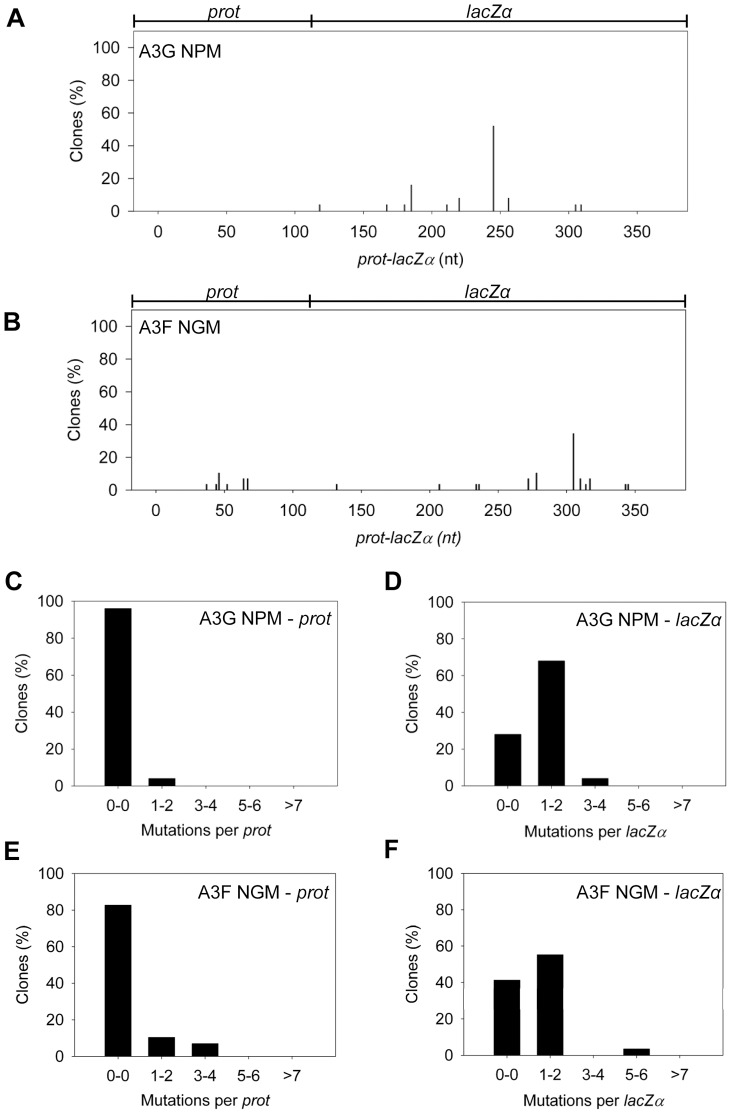
Cytosine deamination-induced mutagenesis by mutant A3G and A3F in a model HIV replication system. (A–B) Spectra of mutations are plotted as the percentage of clones containing a mutation at a particular location (nt) in the 368 nt *prot-lacZ*α construct for (A) A3G NPM or (B) A3F NGM. (C–F) Histograms show the number of mutations per *prot* or *lacZα* region for (C–D) A3G NPM or (E–F) A3F NGM.

Our model predicts that the A3G NPM mutant should be a poor inducer of mutagenesis during (−)DNA synthesis due the decreased ability of this mutant to slide on ssDNA (Figure 6). In agreement with the model, the A3G NPM induced mutagenesis poorly in the model HIV replication system (Figure 8A), similar to A3F (Figure 3B), but in contrast to wild-type A3G (Figure 3A). The A3G NPM mutant had a mutation frequency in the HIV replication assay (Table 2, 0.29×10^−2^ mutations/bp), which was 9-fold less than wild-type A3G (Table 2, 2.63×10^−2^ mutations/bp). The spectrum and sequence analysis demonstrated that the sparse mutations induced by A3G NPM were still in 5′GG or 5′GGG contexts, but that much fewer occurred (Figure 8A and Table S2). The A3G NPM mutant rarely induced mutations in the *prot* (Figure 8C) and mutations in the *lacZα* region were less than A3F (compare Figure 8D and Figure 3F). Notably, the A3G forms had a 100- (A3G) to 40- (A3G NPM) fold greater specific activity than A3F (Table 4). However, since A3G NPM and A3F similarly induced less mutations (Figure 8A and Figure 3B) than A3G (Figure 3A), the data indicated that the ssDNA searching mechanism, but not the specific activity was a primary determining factor in levels of A3-induced mutagenesis.

Our model, which is based on mutagenesis data from A3G, predicted that the mutation frequency of A3F NGM should increase in comparison to A3F. However, despite the A3F NGM mutant being able to slide (Figure 7), we found that A3F NGM remained inefficient at inducing mutagenesis in the *in vitro* HIV replication assay (Figure 8B). The induced mutagenesis of A3F NGM (Table 2, 0.34×10^−2^ mutations/bp) was more similar to A3F than A3G (Table 2). This could be due to A3F NGM sliding being ∼2-fold less efficient than A3G (compare Figure 7A–B and Figure 1B–C) or that the recovery of sliding alone in A3F is not sufficient for increasing the levels of mutagenesis. The latter possibility suggested another determining factor specific to A3F may affect its mutagenic ability. Namely, A3F NGM retained two distinct properties of A3F, the formation of tetramers and higher order oligomers (data not shown) and an increase in processivity with increasing distance between deamination motifs (Figure 7 and data not shown). Therefore, we propose that the jumping mechanism of A3F that is retained in A3F NGM and distinct from that of A3G is detrimental to efficient mutagenesis and remains as such even the presence of sliding movements. With this being considered, the contributing factors to the efficiency of A3-induced mutagenesis is not only the balance between sliding and jumping, as exemplified by A3G, but also the type of jumping movements, as exemplified by A3F. The A3F NGM mutant also retained the 5′TTC specificity characteristic of A3F and induced similar mutations in HIV *prot* (Table S2).

### A3 processive scanning mechanism determines ability to restrict HIV in single-cycle replication assays

The biochemical data support the hypothesis that the processive scanning mechanism of the A3 enzyme can determine its mutagenic potential during reverse transcription. However, the *in vitro* HIV replication assay used in our experiments cannot account for how the HIV capsid environment may influence A3 enzyme-induced mutagenesis. Therefore, we used a single-cycle replication assay to test whether mutagenesis induced in the *prot* of HIV*Δvif* proviral DNA by the deamination activity of A3G, A3F and their mutant derivatives would recapitulate the results of A3-induced *prot* mutagenesis in the model HIV replication assay. In agreement with the biochemical data, in the HIV *Δvif* proviral DNA the A3G-induced mutations/kb were 6- to 8-fold higher than those of A3F, A3G NPM or A3F NGM (Table 5). Upon analysis of codon changing mutations, we found that the A3G hotspot in the *prot* was the Trp 42 codon, which was mutated to a stop codon in all clones containing a mutation, except one clone (Table S3). Clones mutated by A3G-catalyzed deaminations also contained other inactivating mutations such as G51R/E or G86R (Table S3). In regards to hotspots, the data were similar for A3G NPM, although fewer mutations were recovered (Table 5 and Table S3). These data supported our biochemical data in which the decrease in sliding by A3G NPM in comparison to A3G resulted in a decrease of mutagenic potential (compare Figure 3A and Figure 8A). The *prot* clones exposed to A3F or A3F NGM had mutations that at best resulted in partial inhibition of protease activity, e.g., D30N or M46I, and none that resulted in complete inactivation of protease activity (Table S3). It is interesting that A3F NGM induced ∼1.3-fold more mutations/kb than A3F, suggesting that there was a slight positive effect of the A3F NGM sliding ability on mutagenesis (Table 5). Overall, the *prot* sequencing data from HIV *Δvif* proviral clones was consistent with the conclusions from the *in vitro* model HIV replication assay and many deamination hotspots were common between the two assays (compare Tables S2 and S3). Differences may have resulted from different temporal dynamics of reverse transcription [59] and that the *in vitro* assay used a smaller segment of the *prot* gene. The observation from *in vitro* data that the 5′TTC motif was less able to cause inactivating mutations than the 5′CCC motif was consistent with HIV *Δvif* proviral DNA exposed to A3F or A3G (Table S3). Not only was the 5′CCC motif able to cause more inactivating mutations by overlapping with the Trp codon (5′TGG), which results in a stop codon, as previously observed [18], but also because it was more likely to cause nonconservative mutations in comparison to the 5′TTC motif (Table S3 and Ref [44]).

**Table 5:** Analysis of A3-induced mutagenesis of *prot* DNA from integrated HIV*Δvif*.

Enzyme	Base pairs sequenced	Total number of G→A mutations	G→A mutation frequency (mutations/kb)
A3G	6318	37	5.9
A3F	7371	5	0.7
A3G NPM	7020	7	1.0
A3F NGM	9126	8	0.9

**Figure 9:**
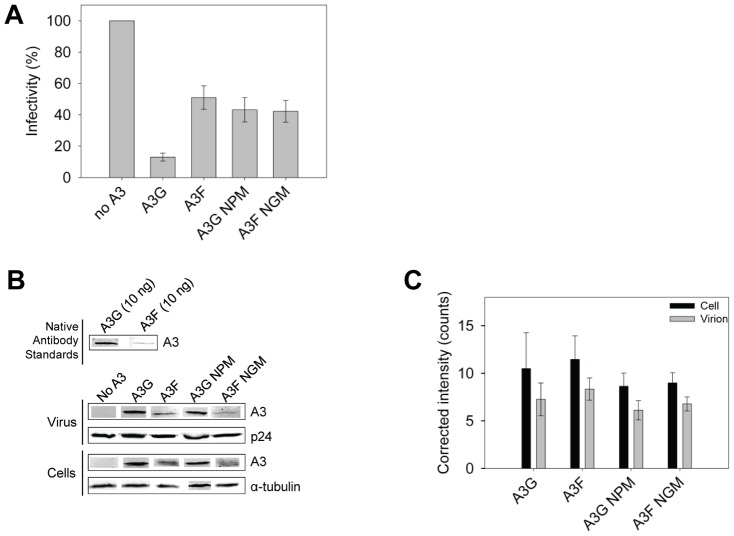
A3 enzyme processivity influences the HIV*Δvif* restriction efficiency. (A) Virus infectivity was measured by eGFP expression in 293T cells infected with HIV*Δvif* that was produced in the absence or presence of A3G, A3F, A3G NPM or A3F NGM. Results normalized to the no A3 condition are shown with the Standard Deviation of the mean calculated from at least three independent experiments. (B–C) Quantitative immunoblotting was used to determine the levels of A3G, A3F, A3G NPM, or A3F NGM expressed in cells and encapsidated into HIV*Δvif* virions. (B) The detection capabilities of antibodies to A3G (Apo C17, NIH AIDS Reagent Program) or A3F (C-18, NIH AIDS Reagent Program) was determined by detecting 10 ng of purified A3G or A3F with a 1/1000 dilution of the appropriate antibody. At this dilution, the antibody to A3F was found to be 9-fold less sensitive than the antibody to A3G. This data was used as a correction factor during quantitation of blots (see Materials and Methods). The loading control for cell lysates was α-tubulin and for virions was p24. (C) Blots from at least three independent experiments were analyzed using Odyssey software to determine the band intensity in the cell lysate (black bars) or virions (gray bars). The values were then corrected to account for the antibody sensitivity (see Materials and Methods). Loading controls were confirmed during quantification to not be significantly different (data not shown). The error bars represent the Standard Deviation of the mean. A t-test determined that there were no significant differences in enzyme expression or encapsidation.

The impact of A3G- and A3F-induced mutations on the infectivity of the proviral DNA was also examined using the eGFP reporter gene contained in the HIV pNL4-3 *Δvif* construct. Consistent with sequencing data from the *prot* region, the eGFP reporter gene of the integrated provirus from the same assays was inactivated 3- to 4-fold more in HIV *Δvif* virions exposed to A3G in comparison to A3F, A3G NPM or A3F NGM (Figure 9A). To ensure this was not due to differences in encapsidation efficiency between these A3 enzymes we conducted quantitative immunoblotting on virions and cell lysates. Since we had transfected untagged A3 enzymes for these experiments to avoid the potential effects a tag may have on processivity (Figure S7), we initially standardized the antibodies for native A3G and A3F. Using equivalent amounts of purified protein and antibody dilutions, we determined that the antibody to A3F was 9-fold less sensitive than the antibody to A3G (Figure 9B). As a result, we used this as a correction factor in the calculated amounts of these enzymes in virions and cells (Figure 9C). The immunoblot results demonstrated that A3G and A3F were expressed in 293T cells and encapsidated into *Δvif* virions to a similar level (Figure 9B–C). Therefore, the data support that there is a *bone fide* difference in the inherent mutagenic abilities of A3G and A3F. We also confirmed that A3G and its NPM mutant and A3F and its NGM mutant were expressed in cells and encapsidated in virions similarly (Figure 9B–C) enabling comparisons to be made between the mutant and wild-type forms of the enzymes. The analysis of A3G or A3F mutants from single-cycle infectivity assays was consistent with biochemical data. The A3G NPM mutant that had diminished sliding ability was less able to restrict HIV replication than A3G (Figure 9A, 3-fold). A3F NGM was able to decrease HIV*Δvif* infectivity 10% more than A3F, suggesting a slight positive effect of its sliding ability, but this was not statistically significant (Figure 9A). These data provided evidence that the processive scanning mechanism of the A3 enzyme influences the capacity to restrict HIV in a single cycle of replication. The disparity in HIV restriction efficiency was confirmed to be due to differences in mutational load by sequencing the HIV*Δvif* integrated provirus *eGFP* reporter gene (Figure S8).

## Discussion

Reports have demonstrated that A3F is less effective than A3G at restricting HIV replication and leaves less of a mutational footprint [22], [23], [24], [29]. This could be due to many reasons such as differences in mRNA/protein expression levels [22], [31], virion encapsidation levels [4], [33], deamination site preference [4], [18], or the inherent biochemical characteristics of the enzymes that govern deamination activity during proviral DNA synthesis. There is no consensus in the literature regarding whether any of the variables determined by cellular conditions, e.g., mRNA expression levels, create disparity between A3F and A3G HIV restriction activities. In addition, other reports have found an equal capacity of A3F and A3G to restrict HIV [3], [5], [6], [7], [20], [21], [30]. To account for these differences in the literature we undertook a biochemical characterization of A3F in comparison to A3G. The data have enabled us to form a biochemical model to account for cell-based observations and propose that the processive DNA scanning mechanism and the preferred deamination motif of A3 deoxycytidine deaminases are determinants of HIV restriction efficiency.

The data support the hypothesis that a balanced sliding and jumping scanning mechanism is a major contributor to efficient restriction of HIV [34] and A3F has less potential to restrict HIV because it does not slide and uses a jumping translocation mechanism that is different than A3G (Figure 1 and Figure 2). Analysis of A3G and A3F mutants further support the model in which the mechanism that the enzymes scan DNA and not their specific activity can fully account for differences observed in HIV restriction (Table 4, Figure 3, Figure 8, and Figure 9). In addition, A3F-induced mutations in preferred 5′TTC/5′TC motifs were less efficient at inducing gene inactivation than the preferred A3G 5′CCC/5′CC motifs, similar to what was identified for A3A (prefers 5′TTC/5′TC) [44], adding another distinction in the mutagenic ability of A3F (Tables S2 and S3).

However, the data cannot support that A3F has no effect on HIV since it is suppressed by Vif [6], but there is evidence that the restriction abilities are distinct from A3G in regards to mutagenic load, selection pressure on HIV and contribution of deamination-independent HIV restriction [24], [29], [60], [61], [62], [63]. It was initially recognized by Zennou and Bieniasz that per mutation, A3G could cause a much larger decrease in HIV infectivity than A3F [4]. This early study on A3F [4] was in contrast to other early studies published showing A3F was similar in effectiveness to A3G [3], [5], [6], [7], [30]. Such incongruent data still remains in the literature [21], [22], [23], [24], [29] and may be due to different experimental systems. Specific to our data, we observed that the HIV*Δvif* retained 51% infectivity in the presence of A3F and 13% infectivity in the presence of A3G, suggesting that A3F is not as effective as A3G at restricting HIV (Figure 9A). However, Albin *et al.* found that over multiple replication cycles, A3F restricted HIV replication similarly to A3G and selected for Vif mutant revertants [61]. It may be that A3G requires only one exposure to HIV for high level restriction compared to A3F that may require multiple cycles for strong HIV restriction, but the end point is the same. Importantly, multiple infection rounds more closely mimics how A3 enzymes would interact with HIV *in vivo*. Nonetheless, our data propose that the mechanism by which A3G and A3F reach this end is different and that A3F has the potential to cause more sequence diversification of HIV than A3G. This idea is supported by Chaipan *et al.* that found that A3F suppressed HIV in multiple rounds of replication but required a longer period of exposure to HIV before the level of suppression reached that of A3G [24]. This is consistent with our sequence data from the *prot* of integrated proviruses (Table S3). As such, the role of A3F may be to supplement mutagenesis induced by A3G [21], [29] since their effects have been shown to be additive [5] or be distinct from A3G and perhaps rely on a deamination-independent mechanism, such as inhibition of reverse transcription and integration [60], [63], [64]. A3F has been reported to exert a larger deamination-independent inhibition of HIV replication than A3G, but this is not as effective as deamination-mediated restriction of HIV [60], [63].

To characterize the mechanism by which A3 enzymes induce mutagenesis we studied the A3G NPM mutant. The A3G NPM mutant demonstrated that the scanning mechanism on DNA and not specific activity is a primary determinant in mutation induction during reverse transcription. Since A3F had a lower specific activity than A3G (Table 4), it could be argued that this was contributing to the lower level of induced mutagenesis (Figure 3). However, the A3G NPM mutant, which had decreased sliding in comparison to wild-type A3G (compare Figure 1 and Figure 6), retained a specific activity more similar to that of A3G than A3F (Table 4), but induced a very low level of mutagenesis (Figure 8A, C–D) and decreased HIV infectivity only 2-fold versus A3G that decreased HIV infectivity 8-fold (Figure 9A). These data suggest that specific activity is not a determinant in the ability to cause mutations during reverse transcription and is supported by previous data in which the specific activity of the enzyme was inconsequential during reverse transcription [43]. This appears to be because the activity of the enzyme during reverse transcription is instead determined by factors such as (−)DNA synthesis and RNaseH activity [43]. The A3G NPM mutant further confirmed that the determinants within the NTD for sliding involve residues near A3G predicted helix 6 and that this is distinct from the determinant for jumping (Figure 6D–E), which in A3G is loop 7 (Figure S6). Of note, the NPM motif is predicted to be at the end of NTD helix 6, which is a connection point between the NTD and the CTD domains [65], [66]. Through amino acid sequence alignment we identified that A3D is the only other double domain A3 deaminase to contain an NPM motif at the end of predicted helix 6, suggesting that A3D would also lack sliding movements while scanning ssDNA, similarly to A3F. Some specific residues within helix 6 have previously been shown to affect specific activity [34], [66], possibly because of structural changes in the connection between the NTD and CTD that can affect the catalytic activity of the CTD or DNA binding affinity. Insertion of the NPM motif into A3G immediately after the predicted helix 6 ends (Figure 6A) did not cause a large disruption in structure based on CD spectra (data not shown), but did result in a ∼3-fold decrease in specific activity (Table 4) and ∼2-fold increase in binding affinity for ssDNA (Table 1).

To confirm a role of the NPM motif in blocking sliding movements we mutated this region in A3F to create an A3F NGM mutant with the hypothesis that removing the rigid proline residue would enable the enzyme to slide on ssDNA and deaminate closely spaced residues. Consistent with the hypothesis, closely spaced residues were processively deaminated by A3F NGM (Figure 7A–B). However, the ability to slide did not enable A3F NGM to induce high levels of mutagenesis similar to A3G *in vitro* (Figure 8) or in a single-cycle infectivity assay (Figure 9A and Table 5). This does not preclude that jumping and sliding are important for inducing mutagenesis in virus infected cells since the A3G NPM mutant that had decreased sliding restricted HIV similarly to A3F in single cycle infectivity assays (Figure 9A). Rather, these data indicated that the ability to slide and jump is necessary, but not sufficient to induce high levels of mutagenesis. The data supported the conclusion that the type of sliding and jumping movements, e.g., distance transversed was also important. Namely, we found that A3F processivity on ssDNA increased with increasing distance between deamination motifs, in contrast to A3G, demonstrating that the average jumping distance of A3F was larger than A3G (Figure 2A–B). This was confirmed with sequence analysis from the model HIV replication assay in which a larger number of deaminations were >20 nt apart for A3F than A3G (Table 3). Thus, the A3F NGM mutant could slide, but was not truly a mimic of A3G. All together, it appears that the sliding and jumping mechanism of A3G is specifically optimal to induce a large number of deaminations during reverse transcription of DNA.

An important note regarding the study of A3F is that we found N-terminally tagged GST-A3F was not processive (Figure S7), despite binding ssDNA with a K_d_ of 46±4 nM. That the binding affinity of GST-A3F was more similar to A3F (Table 1, K_d_ of 20 nM) than A3F CTD (Table 1, K_d_ of 288 nM) indicated that the GST-A3F was able to bind ssDNA with both NTD and CTD domains, despite a lack of processivity. This suggested that the GST tag caused steric hindrance on amino acid determinants for processivity in the NTD. Interestingly, we observed that nonprocessive A3F forms, both A3F CTD and GST-A3F, induced more mutations than wild-type, processive A3F (compare Figure 3 and Figures S9 and S10). We also found that A3A, which is largely nonprocessive, induced slightly less mutations than A3G in the *in vitro* HIV replication assay [44], but more than A3F. Although this initially seems difficult to reconcile, it is consistent with the overall hypothesis that processivity is related to mutagenic potential, since processive A3G is still the most efficient at inducing mutagenesis. It is only that a lack of processivity appears to be better than an “ineffective” processive enzyme such as A3F. This is not due to differences in the assay systems for characterizing processive deaminations on ssDNA oligonucleotides and the model HIV replication assay since addition of NC and RT to the ssDNA oligonucleotides in a deamination reaction did not change our observations regarding A3F CTD processivity (Figure S11). In comparison to the nonprocessive A3F CTD and GST-A3F, processive A3F leaves many potential deamination motifs unmodified (compare Figure 3 and Figures S9 and S10). Although there is inefficiency in the GST-A3F and A3F CTD having to dissociate and reassociate with the substrate many times, the reassociations can be much closer to the previous dissociation resulting in a more thorough search of the DNA. For example, we found that in the model HIV replication assay, 61% of A3F CTD-induced mutations were >20 nt apart in contrast to A3F where 75% of induced mutations were >20 nt apart (Table 3 and data not shown). Since the HIV replication assay is not conducted under single hit conditions, the results emphasize the inefficiency of the searching mechanism used by A3F. Since the binding affinity of A3F for ssDNA is tighter than A3G or A3F CTD (Table 1), it is conceivable that A3F may also have a lack of frequent movements or excursions on the ssDNA that contribute to the inefficient search for deamination motifs. However, resolution of this speculation awaits single-molecule analysis. In sum, the data demonstrated that the interactions of A3F with ssDNA are essentially detrimental to its ability to induce a high mutation frequency.

Our data demonstrate two main points. First, the data provide a biochemical reason for the inefficiency with which A3F-induces mutagenesis of HIV*Δvif* as observed in this report and by others [22], [23], [24] by demonstrating that the processive scanning behavior of A3F is detrimental to its mutagenic potential. The data establish that a balanced sliding and jumping ssDNA scanning mechanism similar to A3G is required for the most efficient induction of HIV mutagenesis. Secondly, the data show that deamination of 5′CCC/5′CC has more gene inactivating potential than 5′TTC/5′TC providing an additional reason for less restriction of HIV by A3F than A3G, in agreement with previous reports [4], [18], [44]. The data does not preclude that A3F can effectively restrict HIV and is in agreement with studies showing that A3F can restrict HIV in multiple rounds of infection [24], [61], but since the number of mutations induced has been correlated to HIV inactivation [45], [67], the data support the interpretation that A3F inactivates HIV less efficiently than A3G in a single round of infection.

## Materials and Methods

### Protein expression and purification

Recombinant baculovirus production for expression of GST-A3G, GST-A3F (NCBI Accession BC038808), GST-A3G CTD (amino acids 197–380), GST-A3F CTD (amino acids 195–373), GST-A3G NPM, GST-A3F NGM or GST-nucleocapsid protein (NC) in *Sf*9 cells was carried out using the transfer vector pAcG2T (BD Biosciences), as previously described [34], [35], [54]. Site directed mutagenesis was used to create the A3G NPM and A3F NGM clones. Cloning primers for A3 enzymes and the site directed mutagenesis primers were obtained from Integrated DNA Technologies and are listed in Table S1. *Sf*9 cells were infected with recombinant virus at a multiplicity of infection (MOI) of 1, except for GST-A3F and GST-A3F CTD which were infected at an MOI of 2. Recombinant baculovirus infected *Sf*9 cells were harvested after 72 h of infection. Cells were lysed in the presence of RNaseA and the proteins (A3G, A3G NPM, A3G CTD, and NC) were purified as described previously [54] to obtain protein that was cleaved from the GST tag and 95% pure. The A3F, A3F NGM, and A3F CTD enzymes were eluted from the glutathione-sepharose resin (GE Healthcare) with the GST tag, as previously described [35]. The samples were then treated with thrombin (Merck Millipore; A3F and A3F NGM, 0.02 U/µL; A3F CTD, 0.10 U/µL) for 2–5 hours at 21°C to cleave the GST tag. A DEAE Fast Flow column (GE Healthcare) was then used to purify the A3F, A3F NGM, and A3F CTD from the GST tag and thrombin. The proteins were loaded in low salt buffer containing 50 mM Tris pH 8.0, 50 mM NaCl, 10% glycerol, and 1 mM DTT. A linear gradient from 50 mM NaCl to 1 M NaCl was used to differentially elute the enzymes. The enzymes eluted at approximately 450 mM NaCl and were 90% pure. The SDS-PAGE gels of the purified A3 enzymes are shown in Figure S12. Protein fractions were stored at −80°C. HIV RT (p66/p51) [68] was generously provided by Dr. Stuart F.J. Le Grice (NCI, National Institutes of Health).

### Size exclusion chromatography

The oligomerization state of A3 enzymes was determined by subjecting 10–15 µg of the purified enzymes to size exclusion chromatography using a 10 mL Superdex 200 (GE Healthcare) resin bed contained in a column with a 0.5 cm diameter and 16 cm height. The running buffer used was 50 mM Tris pH 7.5 and 200 mM NaCl. The Bio-Rad gel filtration standard set was used to generate a standard curve from which molecular masses and oligomerization states were calculated.

### Model HIV replication assay

A3-induced mutagenesis of ssDNA during reverse transcription of an RNA template was measured using an *in vitro* assay, which models reverse transcription from an RNA template and second strand synthesis, and was performed as described previously [34]. Briefly, a synthetic (+)RNA is synthesized that contains a polypurine tract (PPT), 120 nt of the catalytic domain of the HIV protease (*prot*), and *lacZα* (248 nt). The PPT is used as a primer for (+)DNA synthesis and enables synthesis of dsDNA. The *lacZα* serves as a reporter gene for mutations by blue/white screening. The HIV protease gene was obtained by PCR using clone p93TH253.3 obtained through the AIDS Research and Reference Reagent Program, Division of AIDS, NIAID, NIH from Dr. Feng Gao and Dr. Beatrice Hahn [69]. The RNA template (50 nM) was annealed to a 24 nt DNA primer [34] and incubated with NC (1.5 µM), RT (1.2 µM), and dNTPs (500 µM) in RT buffer (50 mM Tris pH 7.4, 40 mM KCl, 10 mM MgCl_2_, 1 mM DTT) in the presence or absence of 200 nM of A3G, A3F, A3G NPM or A3F NGM. Synthesized dsDNA was PCR amplified using Pfu C_x_ Turbo Hotstart (Agilent Technologies) that can use uracils as a template with high fidelity. The amplicons were cloned into a pET-Blue vector backbone that would allow the experimentally synthesized *lacZα* to be used for α-complementation [34]. At least twenty-five mutated clones for each condition tested were analyzed. DNA sequencing was carried out at the National Research Council of Canada (Saskatoon, Saskatchewan). A t-test was used for statistical analysis of sequences.

### Deamination assays

The ssDNA substrates were obtained from Tri-Link Biotechnologies and are listed in Table S1. Deaminations were detected by resolving Fluorescein (F)-labeled DNA that had been treated with Uracil DNA Glycosylase (New England Biolabs) and heated under alkaline conditions on a 10%, 16%, or 20% v/v denaturing polyacrylamide gel, as described previously [35]. The gel type was determined by fragment sizes produced by each substrate. Reactions were carried out under single hit conditions, i.e., <15% substrate usage [46], to ensure that a single ssDNA substrate was interacting with at most a single enzyme. Under these conditions, a processivity factor can be determined by comparing the total number of deaminations occurring at two sites on the same DNA substrate to a calculated theoretical value of the expected deaminations that would occur at those two sites if the deaminations were not processive (see reference [35]). In order to obtain substrate usage within this range under steady-state conditions, the enzyme and DNA concentration were varied based on the enzyme specific activity. More ssDNA was used with A3G to ensure clear observation of all deamination bands despite the large preference for the 5′C. However, the data are not altered with ssDNA concentration (data not shown). For A3G and A3G NPM, 30, 40, or 100 nM enzyme was incubated with 300 or 500 nM fluorescein (F)-labeled ssDNA. For A3F, A3F NGM, and A3F CTD, 100 nM enzyme was incubated with 50 or 100 nM F-labeled ssDNA. For A3G CTD, 1000 nM enzyme was incubated with 500 nM F-labeled ssDNA. Reactions were incubated at 37°C for 1–50 min. Gel pictures were obtained using a Typhoon Trio (GE Healthcare) multipurpose scanner and analysis of integrated gel band intensities used ImageQuant software (GE Healthcare). The specific activity was calculated from single-hit condition reactions by determining the picomoles of substrate used per minute for a microgram of enzyme.

### Steady state rotational anisotropy assays

Steady state fluorescence depolarization (rotational anisotropy) was used to measure enzyme-ssDNA binding affinities using the same F-labeled ssDNA substrates (with cytosines 63 nt apart) that were used for deamination reactions (Table S1). Reactions were 60 µL and contained F-labeled ssDNA (10 nM) in RT buffer and A3G (0–650 nM), A3F (0–80 nM), A3F CTD (0–600 nM), A3G NPM (0–350 nM), or A3F NGM (0–650 nM) were titrated into the reaction. A QuantaMaster QM-4 spectrofluorometer (Photon Technology International) with a dual emission channel was used to collect data and calculate anisotropy. Measurements were made at 21°C. Samples were excited with vertically polarized light at 495 nm (6 nm band pass) and vertical and horizontal emissions were measured at 520 nm (6 nm band pass). Apparent dissociation constants (K_d_) were obtained by fitting to a sigmoidal curve using Sigma Plot 11.2 software.

### Single-cycle infectivity assay

VSV-G pseudotyped HIV pNL4-3 *Δvif* viruses were produced by transfecting 3×10^5^ 293T cells per well in a 6-well plate with Qiagen Polyfect reagent. Specifically, transfections used 1100 ng of pHIV*Δvif* [70], which expresses an eGFP reporter gene and 630 ng of pLTR-G (Addgene), which expresses the VSV-G protein, in the presence or absence of 220 ng of A3G, A3F or A3F NGM or 350 ng of A3G NPM in pcDNA3.1. The transfections used empty pcDNA3.1 to achieve equivalent amounts of DNA. The cotransfection molar ratio of A3 enzymes in pcDNA3.1 to the pNL4-3 *Δvif* was 0.33:1 (A3G, A3F, or A3F NGM) or 0.59:1 (A3G NPM). The A3G (cat# 9952) and A3F (cat # 10100) expression plasmids were obtained from the NIH AIDS Reagent program with C-terminal tags. A stop codon was introduced immediately after the A3G or A3F coding sequence to enable expression of native A3 enzymes. The amino acid sequence of the A3G and A3F clones were identical to those used in biochemical assays. Subsequently, site directed mutagenesis was used to create the A3G NPM and A3F NGM clones. The site directed mutagenesis primers were obtained from Integrated DNA Technologies and are listed in Table S1. Sixteen hours after the transfection, the cells were washed with PBS and the medium replaced. Virus-containing supernatants were collected 48 hours after the media change and filtered through 0.22 µm syringe filters. Virus was quantified by a p24 enzyme-linked immunosorbent assay (QuickTiter Lentivirus Titer Kit, Cell Biolabs Inc.). Target 293T cells were infected at an MOI of 0.5 by spinoculation at 800× *g* for 1 h in the presence of 8 µg/ml of polybrene [71]. Infection levels in 293T cells was determined by flow cytometry by detecting eGFP fluorescence at 48 hours post infection and data were normalized to HIV*Δvif* infections in the absence of A3 enzymes.

### Sequencing of integrated proviral DNA

Infected 293T cells were harvested after 48 h and the DNA was extracted using the Qiagen DNeasy Blood and Tissue kit. DNA was treated with DpnI (New England Biolabs) to remove possible contaminating plasmid DNA and the *prot* (nt 2280-2631) sequences were amplified by PCR using Phusion High Fidelity Polymerase (New England Biolabs). Primers were obtained from Integrated DNA and are listed in Table S1. PCR products were purified and cloned with the Zero Blunt TOPO PCR cloning kit (Invitrogen). DNA sequencing was carried out at the National Research Council of Canada (Saskatoon, Saskatchewan).

### Quantitative immunoblotting

The A3G and A3F enzymes were detected in cell lysates (40 µg total protein) and virions (130 ng of p24) used for single-cycle infectivity assays using antibodies to the native enzymes. For A3G we used the ApoC17 rabbit antiserum (Cat # 10082, NIH AIDS Reagent Program) and for A3F we used the C-18 polyclonal rabbit antibody (Cat # 11474, NIH AIDS Reagent Program). Loading controls for cell lysates (α-tubulin, Sigma) and virions (p24, Cat #3537, NIH AIDS Reagent Program) were detected using mouse monoclonal antibodies. Proteins of interest and loading controls were detected in parallel on the same gel by using the Licor/Odyssey system (IRDye 680-labeled goat anti-rabbit secondary antibody and IRDye 800-labeled goat anti-mouse secondary antibody). Visualization with an Odyssey Infrared Imaging System (Licor) and analysis of bands with Odyssey software enabled intensities of bands to be determined. Analysis of a titration of purified A3G and A3F with their respective antibodies showed that A3F detection was 9-fold less sensitive than A3G detection at a 1/1000 antibody dilution. Further, doubling the amount of antibody to A3F (1/500) resulted in a doubling of the A3F detection sensitivity in comparison to the antibody to A3G (1/1000). Therefore, an appropriate correction factor for the antibody dilution was used to adjust the integrated band intensities of A3F to enable comparison with A3G. Antibodies were used at a dilution of 1/1000 except for A3F or A3F NGM containing cell lysates which required a dilution of 1/500 for detection of A3F or A3F NGM. A t-test was used for statistical analysis.

## Supporting Information

Figure S1 **Binding affinities of A3F and A3G for single-stranded (ss) DNA or double-stranded (ds) DNA.** A3F and A3G binding to fluorescein labeled DNA(10 nM) was monitored with rotational anisotropy. (A) ssDNA as shown in Figure 1D was used as a substrate. A3F binds this ssDNA with a high affinity (apparent K_d_ of 20±1 nM). (B–C) The double stranded region (20 nt) created in Figure 1E was used as a binding substrate for (B) A3F or (C) A3G. (B) A3F was unable to bind the dsDNA to saturation in a concentration range similar to ssDNA. We were unable to concentrate A3F sufficiently to titrate in the necessary amount to saturate the dsDNA substrate. The apparent K_d_ is estimated to be >600 nM. (C) A saturation curve for A3G binding to dsDNA is shown for comparison. A3G binds the dsDNA with an apparent K_d_ of 823±11 nM. Values are an average from at least two independent experiments.
(TIF)

Figure S2 **Processivity of A3F, A3G, and A3G NPM in the presence of a 20 nt RNA/DNA hybrid.** Deamination was tested on a 118 nt ssDNA substrate that contained an internal fluorescein (F)-label and two deamination motifs separated by 63 nt (sketch). A 20 nt complementary RNA was annealed between the two deamination motifs. Single deaminations of the 5′C and 3′C are detected as the appearance of labeled 100- and 81- nt fragments, respectively; double deamination of both C residues on the same molecule results in a 63 nt labeled fragment (5′C & 3′C). (A) A3F, (B) A3G, and (C) A3G NPM are able to processively deaminate the target cytosines by transversing the RNA/DNA hybrid region. A3F is 2-fold more processive than A3G and A3G NPM on this substrate. The measurements of processivity (Processivity factor) and the Standard Deviation of the mean (S.D.) are shown below the gel. The A3F: DNA ratio was 2∶1 and the A3G: DNA and A3G NPM: DNA ratios were 1∶20. Enzyme: DNA ratios were varied due to different specific activities of the enzyme on a given DNA substrate. Values are an average from three independent experiments.
(TIF)

Figure S3 **Analysis of A3F processivity in the presence of a 20 nt dsDNA region and 5′ATC deamination motifs.** Deamination was tested on a 118 nt ssDNA substrate that contained an internal fluorescein (F)-label and two 5′ATC deamination motifs separated by 63 nt (sketch). A 20 nt complementary DNA was annealed between the two deamination motifs. Single deaminations of the 5′C and 3′C are detected as the appearance of labeled 100- and 81- nt fragments, respectively; double deamination of both C residues on the same molecule results in a 63 nt labeled fragment (5′C & 3′C). A3F is able to processively deaminate the target cytosines by transversing the dsDNA region. The measurements of processivity (Processivity factor) and the Standard Deviation of the mean (S.D.) are shown below the gel. The A3F: DNA ratio was 2∶1. Values are an average from three independent experiments.
(TIF)

Figure S4 **Processivity of A3F and A3G on a substrate with deamination motifs separated by 100 nt.** Deamination was tested on a 157 nt ssDNA substrate that contained an internal fluorescein (F)-label and either two 5′TTC (A3F) or 5′CCC (A3G) deamination motifs (sketch). Single deaminations of the 5′C and 3′C are detected as the appearance of labeled 137- and 120- nt fragments, respectively; double deamination of both C residues on the same molecule results in a 100 nt labeled fragment (5′C & 3′C). A3F (left) and A3G (right) are able to processively deaminate the target cytosines. The measurements of processivity (Processivity factor) and the Standard Deviation of the mean (S.D.) are shown below the gel. The A3F: DNA ratio was 1∶1 and the A3G: DNA ratio was 1∶20. Values are an average from three independent experiments.
(TIF)

Figure S5 **Increasing the total concentration of enzyme and substrate does not decrease the processivity of A3G.** Deamination was tested on an 85 nt ssDNA substrate that contained an internal fluorescein (F)-label (yellow star) and two deamination motifs separated by 30 nt (sketch). Single deaminations of the 5′C and 3′C are detected as the appearance of labeled 67- and 48- nt fragments, respectively; double deamination of both C residues on the same molecule results in a 30 nt labeled fragment (5′C & 3′C). The processivity of A3G was not significantly changed when the enzyme: substrate (E∶S) ratio (1∶16) was kept constant, but reaction components increased (3: 50 nM, 30: 500 nM, 60: 1000 nM). The measurements of processivity (Processivity factor) and the Standard Deviation of the mean (S.D.) are shown below the gel. Values are an average from three independent experiments.
(TIF)

Figure S6 **Model of the N-terminal domain (NTD) of A3G.** Model (grey) shows loop 7 and helix 6 (both in red). The amino acids NPM were inserted at the end of predicted helix 6. Zinc atom is a dark grey sphere. The predicted model of A3G NTD was obtained by using the automated SWISS-MODEL program using the homologous A3G CTD (PDB: 3IQS) structure as a template. Figure was made using PyMOL (The PyMOL Molecular Graphics System, Version 1.5.0.5, Schrödinger, LLC.).
(TIF)

Figure S7 **GST-A3F is not processive.** Deamination was tested on an 85 nt ssDNA substrate that contained an internal fluorescein (F)-label (yellow star) and two deamination motifs separated by 30 nt (sketch). Single deaminations of the 5′C and 3′C are detected as the appearance of labeled 67- and 48- nt fragments, respectively; double deamination of both C residues on the same molecule results in a 30 nt labeled fragment (5′C & 3′C). GST-A3F is not processive on this substrate as evidenced by the absence of a double deamination band (5′C & 3′C, 30 nt). The A3F: DNA ratio was 1∶1. A representative gel from three independent experiments is shown.
(TIF)

Figure S8 **Representative eGFP sequences of integrated proviruses.** Representative eGFP sequences from the single-cycle infectivity assay (Figure 9A) are shown. Mutations are in bold. Alignment was made using CLUSTAL W.
(TIF)

Figure S9 **A3F CTD mutagenesis in a model HIV replication system.** (A) Spectrum of mutations are plotted as the percentage of clones containing a mutation at a particular location (nt) in the 368 nt *prot-lacZ*α construct. (B-C) Analysis of the number of mutations induced by A3F-CTD in the (B) *prot* or (C) *lacZα* regions.
(TIF)

Figure S10 **GST-A3F mutagenesis is comparable to A3F CTD in a model HIV replication system.** (A) Spectrum of mutations are plotted as the percentage of clones containing a mutation at a particular location (nt) in the 368 nt *prot-lacZ*α construct. (B–C) Analysis of the number of mutations induced by GST-A3F in the (B) *prot* or (C) *lacZα* regions.
(TIF)

Figure S11 **A3F CTD is not processive in the presence of NC and RT.** Deamination was tested on an 118 nt ssDNA substrate that contained an internal fluorescein (F)-label (yellow star) and two deamination motifs separated by 63 nt (sketch). Single deaminations of the 5′C and 3′C are detected as the appearance of labeled 100- and 81- nt fragments, respectively; double deamination of both C residues on the same molecule results in a 63 nt labeled fragment (5′C & 3′C). A3F CTD is unable to processively deaminate the target cytosines as evidenced by the absence of a 63 nt labeled fragment above background (5′C & 3′C). The A3F CTD: DNA ratio was 2∶1. Three independent experiments were conducted.
(TIF)

Figure S12 **Purity of enzymes.** Purity of the enzymes was assessed by SDS-PAGE and coomassie staining (A3G, A3G NPM, A3F CTD) or Bio-Rad Oriole fluorescent gel stain (A3F, A3F NGM).
(TIF)

Table S1 **Primers and DNA substrates.**
(PDF)

Table S2 **A3-induced mutagenesis in HIV** ***prot*** **region synthesized in a model HIV replication assay.** Protease enzyme activity was inferred from a mutational study carried out by Loeb and colleagues [58], where double plus (++) is active, plus (+) is partially active and minus (−) is inactive in comparison to wild-type protease. Protease inhibitor resistance information is from http://hivdb.stanford.edu. No recorded value is used to indicate that no clones were found with a mutation at that particular site.
(PDF)

Table S3 **A3-induced mutagenesis in integrated proviral HIV-1** ***Δvif prot*** **DNA.** Protease enzyme activity was inferred from a mutational study carried out by Loeb and colleagues [58], where double plus (++) is active, plus (+) is partially active and minus (−) is inactive in comparison to wild-type protease. Protease inhibitor resistance information is from http://hivdb.stanford.edu. No recorded value is used to indicate that no clones were found with a mutation at that particular site.
(PDF)
