# Supplementary figures and images for: Different Mutagenic Potential of HIV-1 Restriction Factors APOBEC3G and APOBEC3F Is Determined by Distinct Single-Stranded DNA Scanning Mechanisms
Source: PLoS Pathog. 2014 Mar 20;10(3):e1004024. doi: 10.1371/journal.ppat.1004024 (PMC3961392; doi:10.1371/journal.ppat.1004024)

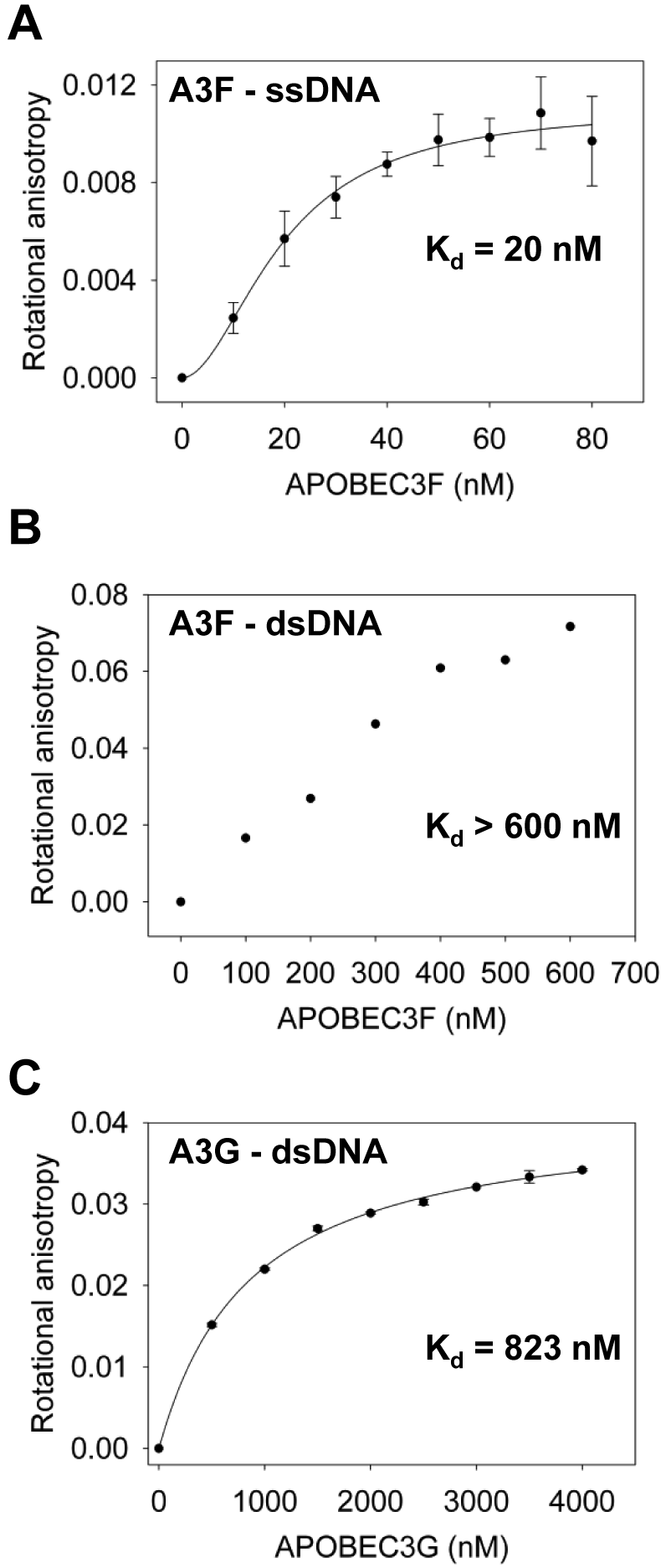

Supplement: Figure S1 — Binding affinities of A3F and A3G for single-stranded (ss) DNA or double-stranded (ds) DNA. A3F and A3G binding to fluorescein labeled DNA(10 nM) was monitored with rotational anisotropy. (A) ssDNA as shown in Figure 1D was used as a substrate. A3F binds this ssDNA with a high affinity (apparent Kd of 20±1 nM). (B–C) The double stranded region (20 nt) created in Figure 1E was used as a binding substrate for (B) A3F or (C) A3G. (B) A3F was unable to bind the dsDNA to saturation in a concentration range similar to ssDNA. We were unable to concentrate A3F sufficiently to titrate in the necessary amount to saturate the dsDNA substrate. The apparent Kd is estimated to be >600 nM. (C) A saturation curve for A3G binding to dsDNA is shown for comparison. A3G binds the dsDNA with an apparent Kd of 823±11 nM. Values are an average from at least two independent experiments. (TIF) [file ppat.1004024.s001.tif]

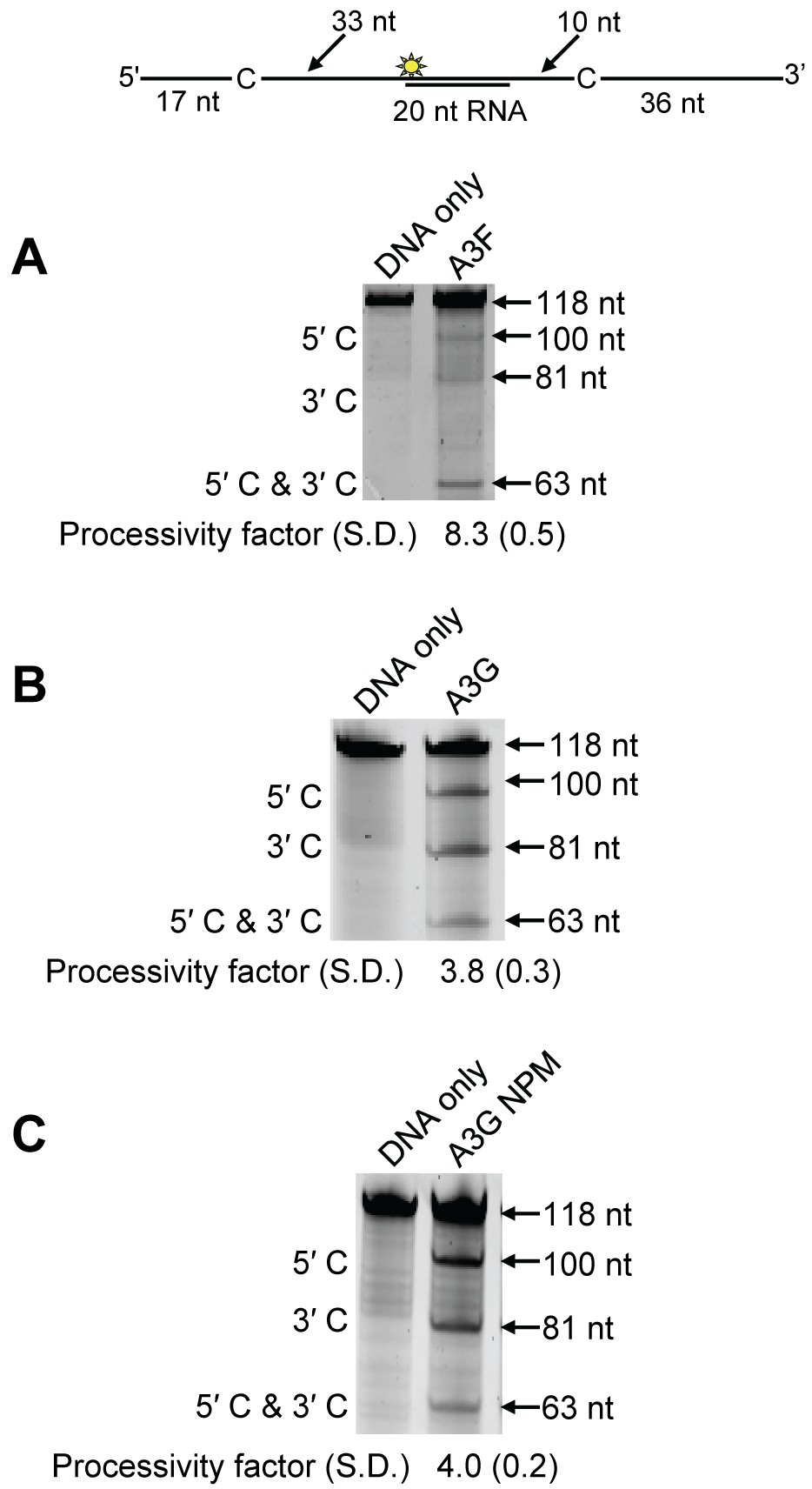

Supplement: Figure S2 — Processivity of A3F, A3G, and A3G NPM in the presence of a 20 nt RNA/DNA hybrid. Deamination was tested on a 118 nt ssDNA substrate that contained an internal fluorescein (F)-label and two deamination motifs separated by 63 nt (sketch). A 20 nt complementary RNA was annealed between the two deamination motifs. Single deaminations of the 5′C and 3′C are detected as the appearance of labeled 100- and 81- nt fragments, respectively; double deamination of both C residues on the same molecule results in a 63 nt labeled fragment (5′C & 3′C). (A) A3F, (B) A3G, and (C) A3G NPM are able to processively deaminate the target cytosines by transversing the RNA/DNA hybrid region. A3F is 2-fold more processive than A3G and A3G NPM on this substrate. The measurements of processivity (Processivity factor) and the Standard Deviation of the mean (S.D.) are shown below the gel. The A3F: DNA ratio was 2∶1 and the A3G: DNA and A3G NPM: DNA ratios were 1∶20. Enzyme: DNA ratios were varied due to different specific activities of the enzyme on a given DNA substrate. Values are an average from three independent experiments. (TIF) [file ppat.1004024.s002.tif]

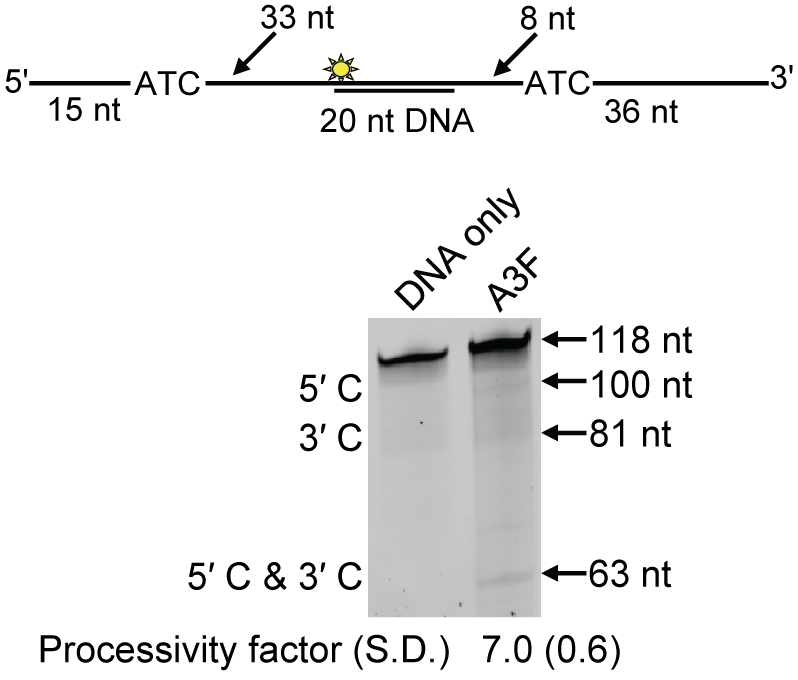

Supplement: Figure S3 — Analysis of A3F processivity in the presence of a 20 nt dsDNA region and 5′ATC deamination motifs. Deamination was tested on a 118 nt ssDNA substrate that contained an internal fluorescein (F)-label and two 5′ATC deamination motifs separated by 63 nt (sketch). A 20 nt complementary DNA was annealed between the two deamination motifs. Single deaminations of the 5′C and 3′C are detected as the appearance of labeled 100- and 81- nt fragments, respectively; double deamination of both C residues on the same molecule results in a 63 nt labeled fragment (5′C & 3′C). A3F is able to processively deaminate the target cytosines by transversing the dsDNA region. The measurements of processivity (Processivity factor) and the Standard Deviation of the mean (S.D.) are shown below the gel. The A3F: DNA ratio was 2∶1. Values are an average from three independent experiments. (TIF) [file ppat.1004024.s003.tif]

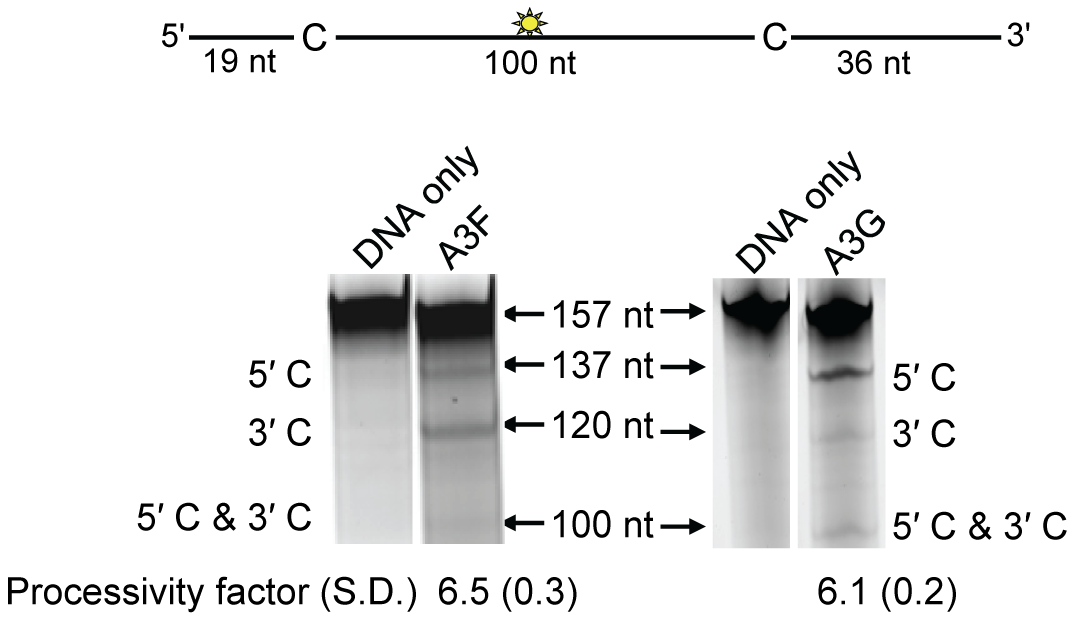

Supplement: Figure S4 — Processivity of A3F and A3G on a substrate with deamination motifs separated by 100 nt. Deamination was tested on a 157 nt ssDNA substrate that contained an internal fluorescein (F)-label and either two 5′TTC (A3F) or 5′CCC (A3G) deamination motifs (sketch). Single deaminations of the 5′C and 3′C are detected as the appearance of labeled 137- and 120- nt fragments, respectively; double deamination of both C residues on the same molecule results in a 100 nt labeled fragment (5′C & 3′C). A3F (left) and A3G (right) are able to processively deaminate the target cytosines. The measurements of processivity (Processivity factor) and the Standard Deviation of the mean (S.D.) are shown below the gel. The A3F: DNA ratio was 1∶1 and the A3G: DNA ratio was 1∶20. Values are an average from three independent experiments. (TIF) [file ppat.1004024.s004.tif]

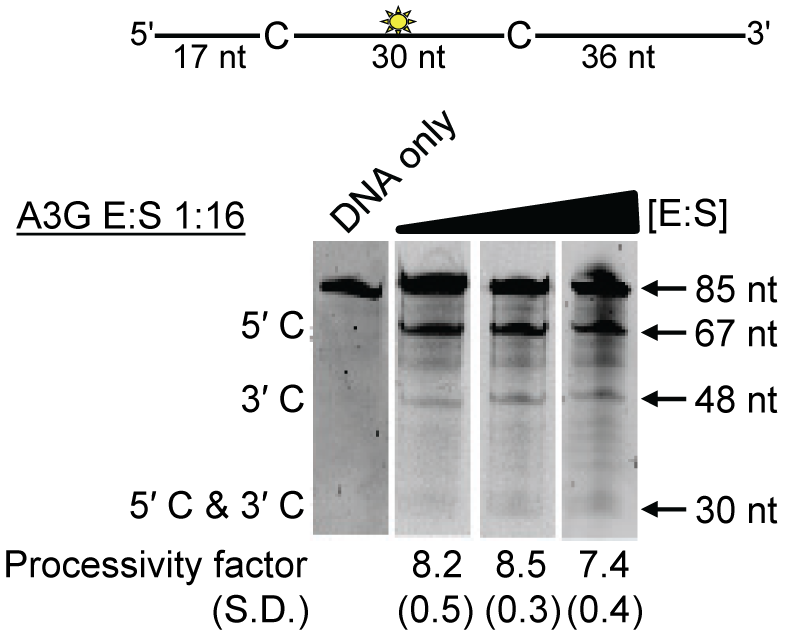

Supplement: Figure S5 — Increasing the total concentration of enzyme and substrate does not decrease the processivity of A3G. Deamination was tested on an 85 nt ssDNA substrate that contained an internal fluorescein (F)-label (yellow star) and two deamination motifs separated by 30 nt (sketch). Single deaminations of the 5′C and 3′C are detected as the appearance of labeled 67- and 48- nt fragments, respectively; double deamination of both C residues on the same molecule results in a 30 nt labeled fragment (5′C & 3′C). The processivity of A3G was not significantly changed when the enzyme: substrate (E∶S) ratio (1∶16) was kept constant, but reaction components increased (3: 50 nM, 30: 500 nM, 60: 1000 nM). The measurements of processivity (Processivity factor) and the Standard Deviation of the mean (S.D.) are shown below the gel. Values are an average from three independent experiments. (TIF) [file ppat.1004024.s005.tif]

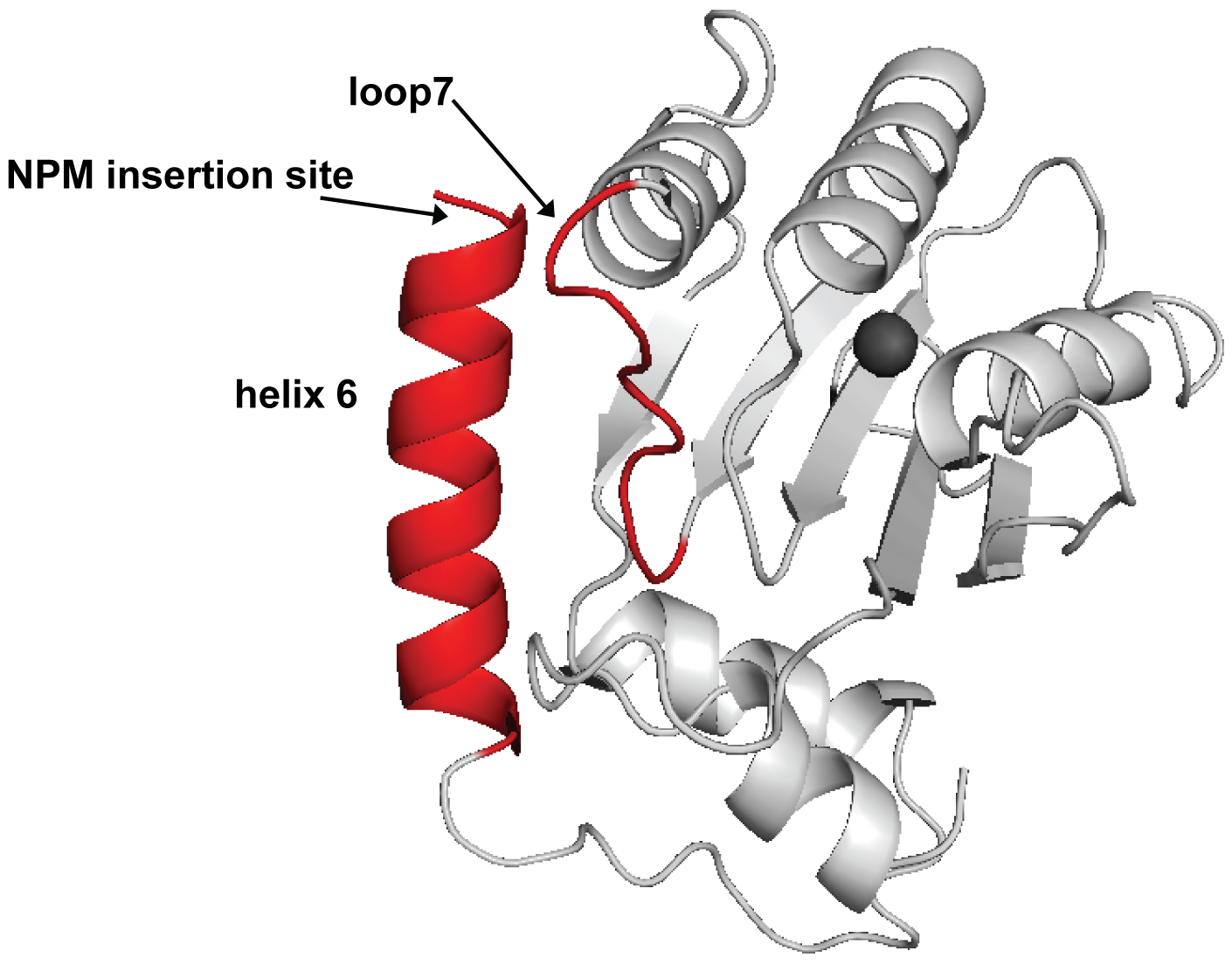

Supplement: Figure S6 — Model of the N-terminal domain (NTD) of A3G. Model (grey) shows loop 7 and helix 6 (both in red). The amino acids NPM were inserted at the end of predicted helix 6. Zinc atom is a dark grey sphere. The predicted model of A3G NTD was obtained by using the automated SWISS-MODEL program using the homologous A3G CTD (PDB: 3IQS) structure as a template. Figure was made using PyMOL (The PyMOL Molecular Graphics System, Version 1.5.0.5, Schrödinger, LLC.). (TIF) [file ppat.1004024.s006.tif]

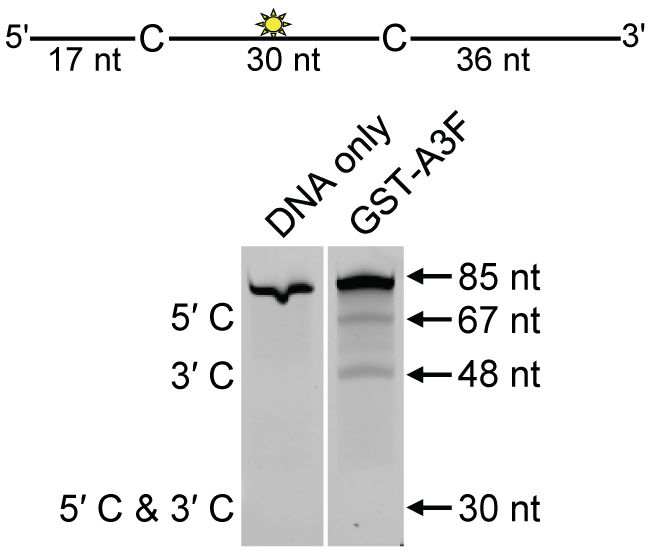

Supplement: Figure S7 — GST-A3F is not processive. Deamination was tested on an 85 nt ssDNA substrate that contained an internal fluorescein (F)-label (yellow star) and two deamination motifs separated by 30 nt (sketch). Single deaminations of the 5′C and 3′C are detected as the appearance of labeled 67- and 48- nt fragments, respectively; double deamination of both C residues on the same molecule results in a 30 nt labeled fragment (5′C & 3′C). GST-A3F is not processive on this substrate as evidenced by the absence of a double deamination band (5′C & 3′C, 30 nt). The A3F: DNA ratio was 1∶1. A representative gel from three independent experiments is shown. (TIF) [file ppat.1004024.s007.tif]

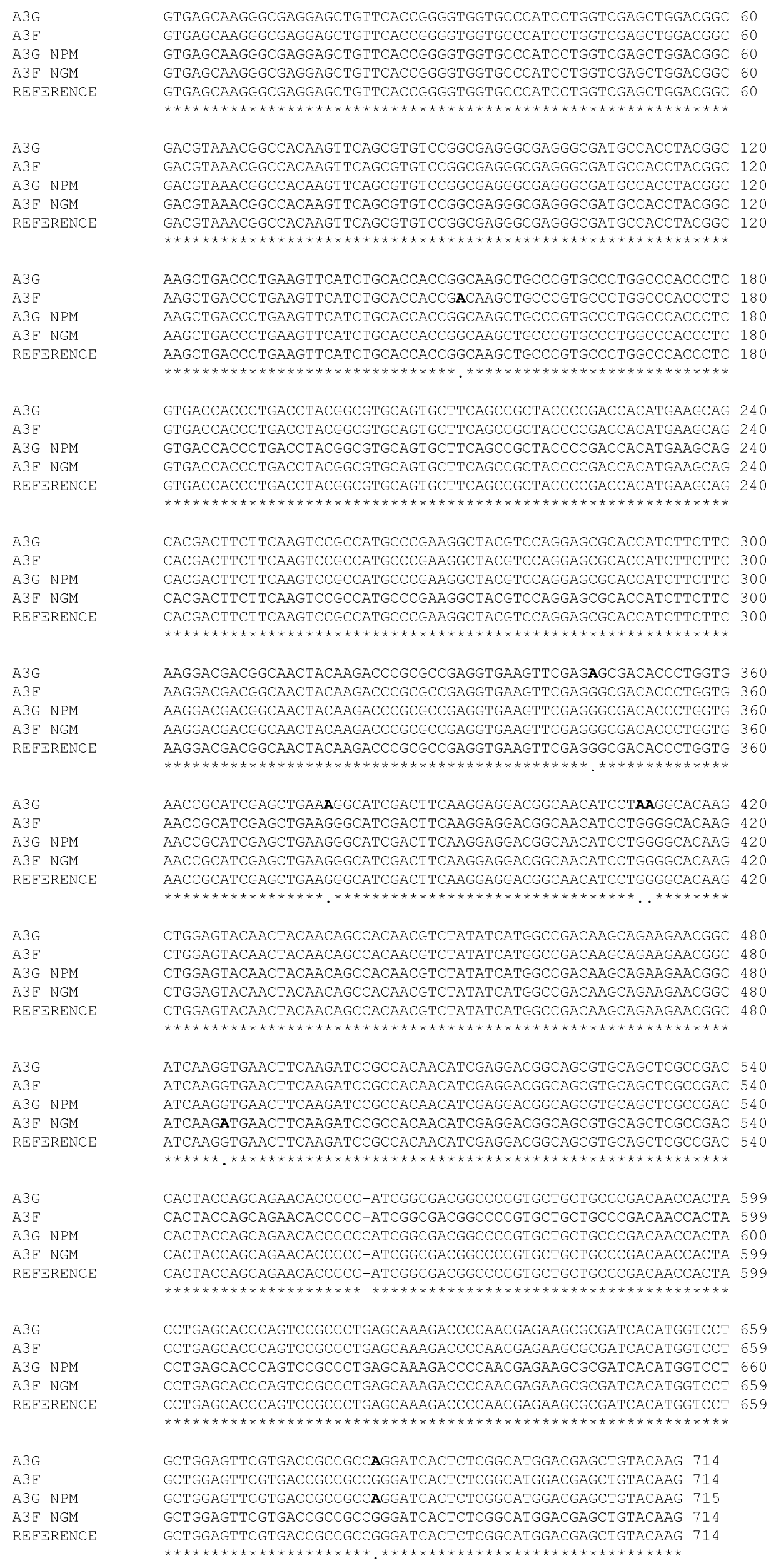

Supplement: Figure S8 — Representative eGFP sequences of integrated proviruses. Representative eGFP sequences from the single-cycle infectivity assay (Figure 9A) are shown. Mutations are in bold. Alignment was made using CLUSTAL W. (TIF) [file ppat.1004024.s008.tif]

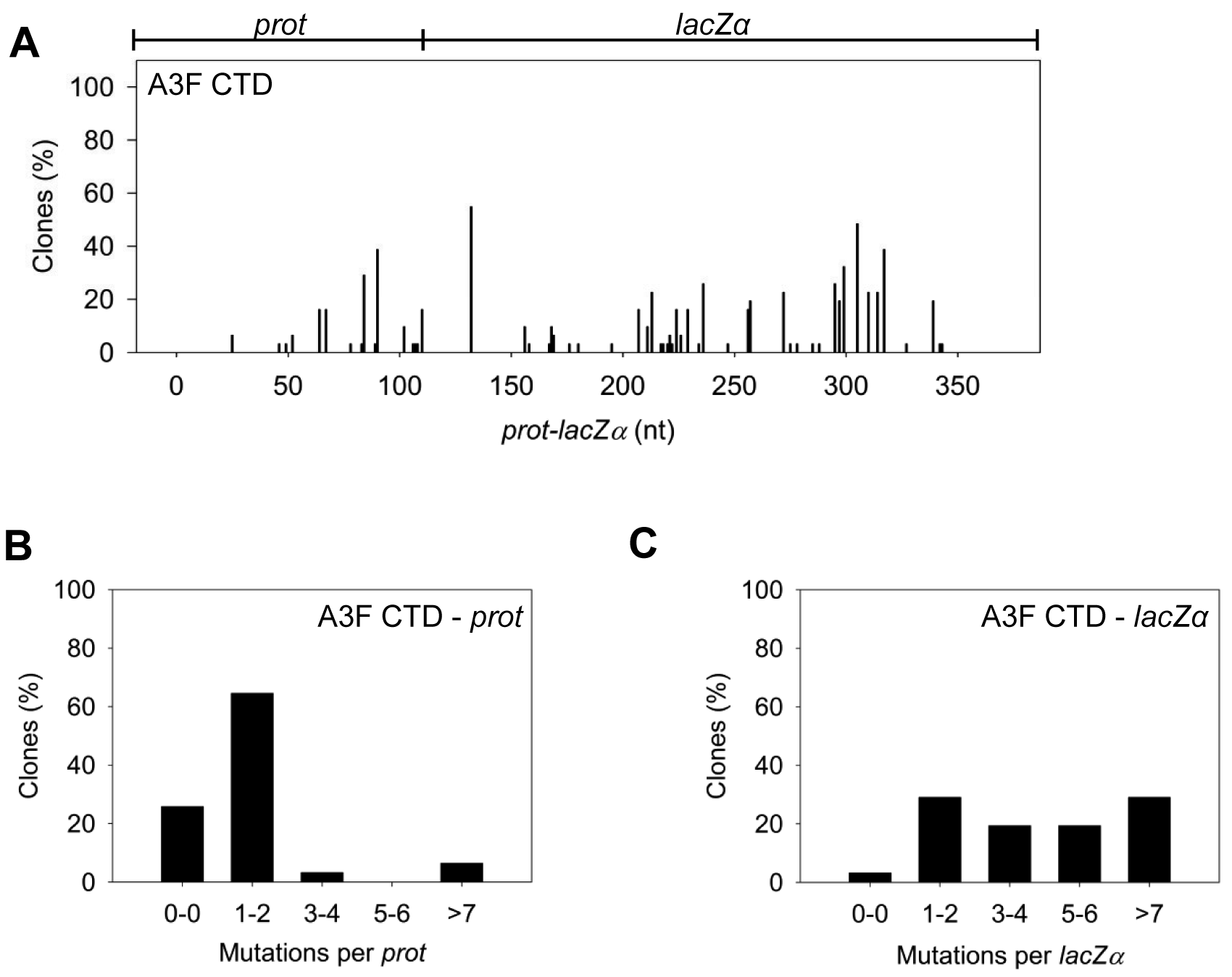

Supplement: Figure S9 — A3F CTD mutagenesis in a model HIV replication system. (A) Spectrum of mutations are plotted as the percentage of clones containing a mutation at a particular location (nt) in the 368 nt prot-lacZα construct. (B-C) Analysis of the number of mutations induced by A3F-CTD in the (B) prot or (C) lacZα regions. (TIF) [file ppat.1004024.s009.tif]

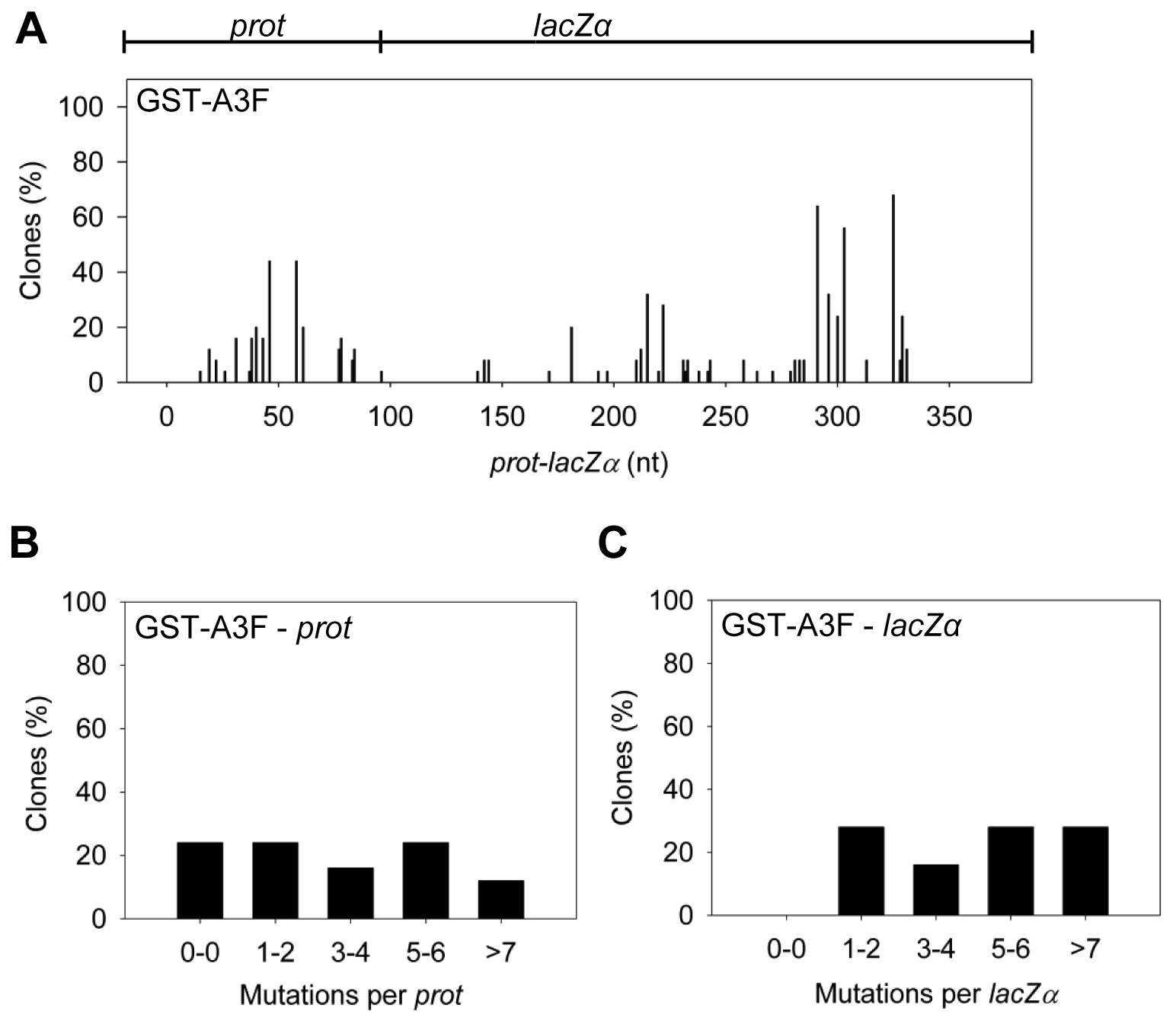

Supplement: Figure S10 — GST-A3F mutagenesis is comparable to A3F CTD in a model HIV replication system. (A) Spectrum of mutations are plotted as the percentage of clones containing a mutation at a particular location (nt) in the 368 nt prot-lacZα construct. (B–C) Analysis of the number of mutations induced by GST-A3F in the (B) prot or (C) lacZα regions. (TIF) [file ppat.1004024.s010.tif]

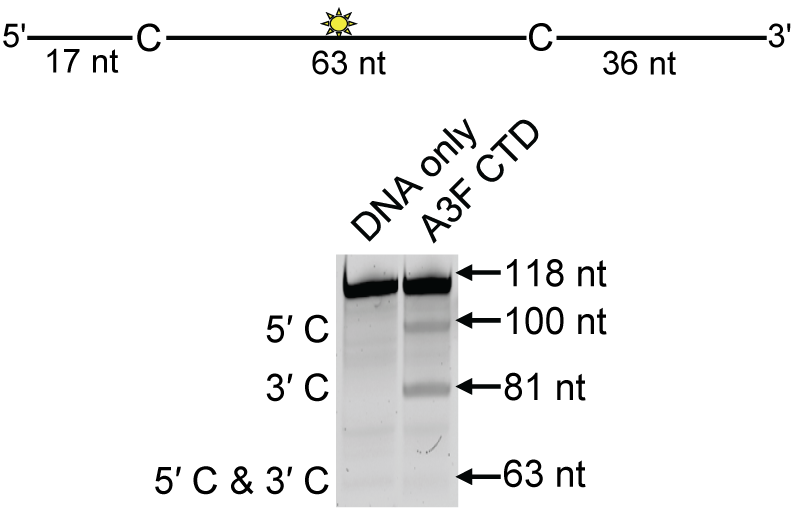

Supplement: Figure S11 — A3F CTD is not processive in the presence of NC and RT. Deamination was tested on an 118 nt ssDNA substrate that contained an internal fluorescein (F)-label (yellow star) and two deamination motifs separated by 63 nt (sketch). Single deaminations of the 5′C and 3′C are detected as the appearance of labeled 100- and 81- nt fragments, respectively; double deamination of both C residues on the same molecule results in a 63 nt labeled fragment (5′C & 3′C). A3F CTD is unable to processively deaminate the target cytosines as evidenced by the absence of a 63 nt labeled fragment above background (5′C & 3′C). The A3F CTD: DNA ratio was 2∶1. Three independent experiments were conducted. (TIF) [file ppat.1004024.s011.tif]

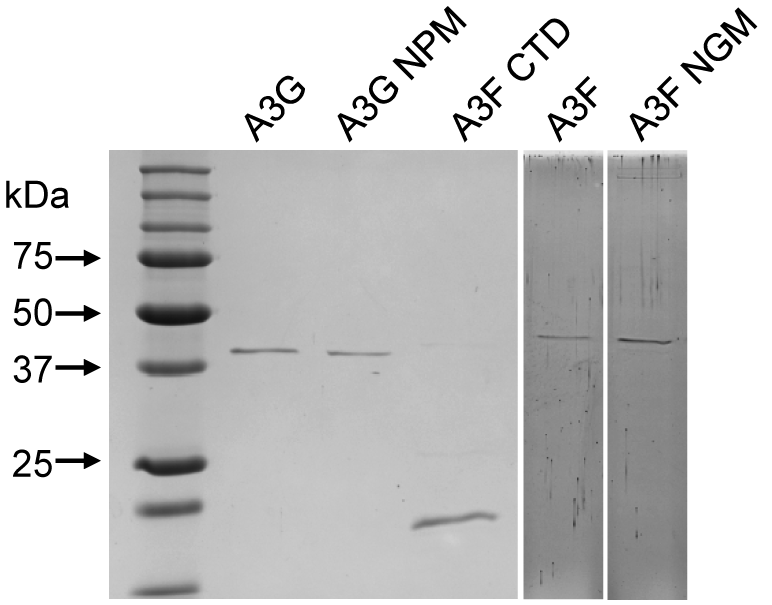

Supplement: Figure S12 — Purity of enzymes. Purity of the enzymes was assessed by SDS-PAGE and coomassie staining (A3G, A3G NPM, A3F CTD) or Bio-Rad Oriole fluorescent gel stain (A3F, A3F NGM). (TIF) [file ppat.1004024.s012.tif]
