# Supplementary material for: Different Mutagenic Potential of HIV-1 Restriction Factors APOBEC3G and APOBEC3F Is Determined by Distinct Single-Stranded DNA Scanning Mechanisms
Source: PLoS Pathog. 2014 Mar 20;10(3):e1004024. doi: 10.1371/journal.ppat.1004024 (PMC3961392; doi:10.1371/journal.ppat.1004024)
Supplement: Table S1 — Primers and DNA substrates. (PDF) [file ppat.1004024.s013.pdf]

Table S1. **Primers and DNA substrates.**

A3F Forward	ATG AAG CCT CAC TTC AGA AAC AC
A3F Reverse	TCA CTC GAG AAT CTC CTG CAG
A3F CTD Forward	ATG TAT CCA CAC ATA TTC TAC TTC
A3F CTD Reverse	TCA CTC GAG AAT CTC CTG CAG CTT G
A3G NPM SDM Forward	GGG GGA GAT TCT CAG AAA CCC GAT GCA CTC GAT GGA TCC ACC
A3G NPM SDM Reverse	GGT GGA TCC ATC GAG TGC ATC GGG TTT CTG AGA ATC TCC CCC
A3F NGM SDM Forward	CTA AAG GAG ATT CTC AGA AAC GGC ATG GAG GCA ATG TAT CCA CAC
A3F NGM SDM Reverse	GTG TGG ATA CAT TGC CTC CAT GCC GTT TCT GAG AAT CTC CTT TAG
A3G Stop SDM Forward	CAG AAT CAG GAA AAC TGA TCT AGA TTT TAC CCA
A3G Stop SDM Reverse	TGG GTA AAA TCT AGA TCA GTT TTC CTG ATT CTG
A3F Stop SDM Forward	CAG GAG ATT CTC GAG TGA AAG GGT CAA GAC AAT TCT G
A3F Stop SDM Reverse	CAG AAT TGT CTT GAC CCT TTC ACT CGA GAA TCT CCT G
Prot Forward	GAC AAG GAA CTG TAT CCT TTA GCT T
Prot Reverse	CTG GTA CAG TCT CAA TAG GAC TAA T
A3G - 5	AAA GAG AAA GTG ATA CCC A{dT-FAM}A CCC ATA GAG TAA AGT TAG TAA GAT GTG TAA GTA TGT TAA
A3F - 5	AAA GAG AAA GTG ATA TTC A{dT-FAM}A TTC ATA GAG TAA AGT TAG TAA GAT GTG TAA GTA TGT TAA
A3G - 14	AAA GAG AAA GTG AGA CCC AAA GAA {dT-FAM}GA AGA CCC AAA TGT TAG AAT TGT TAA TGT GTG TGA TGA TGT TGA
A3F - 14	AAA GAG TTA GTG AGA TTC AAA AT T {dT-FAM}AG AGA TTC AAA TGT TAG ATATGT TAA TGT GTG TGA TGA TGT TGA
A3G - 30	AAA GAG AAA GTG ATA CCC AAA GAG TAA AGT {dT-FAM} AGA TAG AGA GTG ATA CCC AAA GAG TAA AGT TAG TAA GAT GTG TAA GTA TGT TAA
A3F - 30	AAA GAG AAA GTG ATA TTC AAA GAG TAA AGT {dT-FAM} AGA TAG AGA GTG ATA TTC AAA GAG TAA AGT TAG TAA GAT GTG TAA GTA TGT TAA
A3G - 63	GAA TAT ATG TTG AGA CCC AAA GTA ATG AGA GAT TGA {dT-FAM} TAG ATG AGT GTA ATG TGA TAT ATG TGT ATG AAA GAT ATA AGA CCC AAA GAG TAA AGT TGT TAA TGT GTG TAG ATA TGT TAA
A3F - 63 (TTC)	GAA TAT AGT TTT TAG TTC AAA GTA AGT GAA GAT AAT {dT-FAM} TAG AGA GTT GTA ATG TGA TAT ATG TGT ATG AAA GAT ATA AGA TTC AAA GAG TAA AGT TGT TAA TGT GTG TAG ATA TGT TAA

Table S1 (continued). Primers and DNA substrates

A3F - 63 (ATC)	GAA TAT ATG AGT TGA ATC AAA GTA ATG AGA GAG AAT {dT-FAM} TAG ATG AGT GTA ATG TGA TAT ATG TGT ATG AAA GAT ATA AGA ATC AAA GAG TAA AGT TGT TAA TGT GTG TAG ATA TGT TAA
Complementary RNA to A3F/A3G - 63	rCrUrU rUrCrA rUrArC rArCrA rUrArU rArUrC rArC
Trap DNA	AAA GAG AAA GTA ATA AGG AAA GAG TAA AGT ATA TTC AAA TAA ACA ATC ATT CTA CAC ATT CAT ACA ATT
A3G - 100	GGA GAT AGA TTA GAA TAC CC A AAA ATG AAT AAA AAG TGT AGT TGA ATG TAG AAA AGT GGT TAT TGA ATG ATA AGG ATG GAT GGA A{dT-FAM}G ATA TGA AAT GGA GAT AGT GTA GAT GAA AAG ACC CAA AAT GTA GTA AGT AGT TTA AGA ATA GGA GAG TAG T
A3F - 100	GGA GAT AGA TTA GAA TA TTC A AAA AAATAT AAA AAG TGT AGA ATG GTG TAG AAA AGT GGT TAT TGA ATG ATA AGG ATG GAT GGA A{dT-FAM}G ATA TGA AAT GGA GAT AGT GTA GAT GTT AAG ATT CAA AAT GTA GTA AGT AGT TTA AGA ATA GGA GAG TAG T
