# Supplementary material for: Different Mutagenic Potential of HIV-1 Restriction Factors APOBEC3G and APOBEC3F Is Determined by Distinct Single-Stranded DNA Scanning Mechanisms
Source: PLoS Pathog. 2014 Mar 20;10(3):e1004024. doi: 10.1371/journal.ppat.1004024 (PMC3961392; doi:10.1371/journal.ppat.1004024)
Supplement: Table S2 — A3-induced mutagenesis in HIV prot region synthesized in a model HIV replication assay. Protease enzyme activity was inferred from a mutational study carried out by Loeb and colleagues [58], where double plus (++) is active, plus (+) is partially active and minus (−) is inactive in comparison to wild-type protease. Protease inhibitor resistance information is from http://hivdb.stanford.edu. No recorded value is used to indicate that no clones were found with a mutation at that particular site. (PDF) [file ppat.1004024.s014.pdf]

Table S2. **A3-induced mutagenesis in HIV *prot* region synthesized in a model HIV replication assay.** Protease enzyme activity was inferred from a mutational study carried out by Loeb and colleagues [58], where double plus (++) is active, plus (+) is partially active and minus (–) is inactive in comparison to wild-type protease. Protease inhibitor resistance information is from http://hivdb.stanford.edu. No recorded value is used to indicate that no clones were found with a mutation at that particular site.

Protease Amino Acid Position	Nucleotide change	Amino Acid change	Predicted protease activity	Protease inhibitor resistance	Mutated A3F clones (%)	Mutated A3G clones (%)	Mutated A3G NPM clones (%)	Mutated A3F NGM clones (%)
19	CTG→CTA	L→L	++			7		
21	GAA→AAA	E→K	+		4			
22	GCT→ACT	A→T	-		4	3		
25	GAT→AAT	D→N	++		4	7		3
27	GGA→GAA<br>GGA→AGA	G→E<br>G→R	-<br>-			3<br>10		3
28	GCA→ACA	A→T	-		4			10
29	GAT→AAT	D→N	-					
30	GAT→AAT	D→N	+	Yes	4	10		3
34	GAA→AAA	E→K	++		11			7
35	GAT→AAT	D→N	+		4			7
38	TTG→TTA	L→L	++					
40	GGG→GAG<br>GGG→AGG<br>GGG→GAA<br>GGG→GGA	G→E<br>G→R<br>G→E<br>G→G	-<br>-<br>-<br>+			7<br>13		
42	TGG→TAG<br>TGG→TGA<br>TGG→TAA	W→STOP<br>W→STOP<br>W→STOP	-<br>-<br>-		4<br>4	17		
46	ATG→ATA	M→I	+	Yes	11			
48	GGG→AGG<br>GGG→AGA<br>GGG→GAG<br>GGG→GGA	G→R<br>G→R<br>G→E<br>G→G	++<br>++<br>+<br>++			13<br>3<br>3<br>7		
49	GGA→AGA<br>GGA→GAA	G→R<br>G→E	-<br>-			3		
51	GGA→AGA<br>GGA→AAA<br>GGA→GAA	G→R<br>G→K<br>G→E	-<br>-<br>-		 4<br>4	33<br>7<br>3		
52	GGT→AGT	G→S	-		4	47	4	
