# Supplementary material for: Different Mutagenic Potential of HIV-1 Restriction Factors APOBEC3G and APOBEC3F Is Determined by Distinct Single-Stranded DNA Scanning Mechanisms
Source: PLoS Pathog. 2014 Mar 20;10(3):e1004024. doi: 10.1371/journal.ppat.1004024 (PMC3961392; doi:10.1371/journal.ppat.1004024)
Supplement: Table S3 — A3-induced mutagenesis in integrated proviral HIV-1 Δvif prot DNA. Protease enzyme activity was inferred from a mutational study carried out by Loeb and colleagues [58], where double plus (++) is active, plus (+) is partially active and minus (−) is inactive in comparison to wild-type protease. Protease inhibitor resistance information is from http://hivdb.stanford.edu. No recorded value is used to indicate that no clones were found with a mutation at that particular site. (PDF) [file ppat.1004024.s015.pdf]

Table S3. A3-induced mutagenesis in integrated proviral HIV-1△*vif prot* DNA.

Protease enzyme activity was inferred from a mutational study carried out by Loeb and colleagues [58], where double plus (++) is active, plus (+) is partially active and minus (–) is inactive in comparison to wild-type protease. Protease inhibitor resistance information is from http://hivdb.stanford.edu. No recorded value is used to indicate that no clones were found with a mutation at that particular site.

Protease Amino Acid Position	Nucleotide change	Amino Acid change	Predicted protease activity	Protease inhibitor resistance	Mutated A3F clones	Mutated A3G clones	Mutated A3G NPM clones	Mutated A3F NGM clones
20	AAG→AAA	K→K	++			2	1	
21	GAA→AAA	E→K	++		1			
30	GAT→AAT	D→N	+	Yes	1	1		
34	GAA→AAA	E→K	++		1	1		2
35	GAA→AAA	E→K	+					1
36	ATG→ATA	M→I	+		1			3
41	AGA→AAA	R→K	++		1	1		1
42	TGG→TAG	W→STOP	-			5	1	
	TGG→TGA	W→STOP	-			1		
46	ATG→ATA	M→I	+	Yes		1		1
48	GGG→AGA	G→R	++			1		
	GGG→AGG	G→R	++			1		
	GGG→AAA	G→K	++			1		
	GGG→GGA	G→G	++			1		
51	GGA→AGA	G→R	-			3	2	
	GGA→GAA	G→E	-			1		
52	GGT→AGT	G→S	-			2		
57	AGA→AAA	R→K	++			1		
60	GAT→AAT	D→N	++			1		
65	GAA→AAA	E→K	-			2		
73	GGT→AGT	G→S	++			2		
78	GGA→AGA	G→R	-			1		
86	GGA→AGA	G→R	-			3	2	
87	AGA→AAA	R→K	++			1		
90	TTG→TTA	L→L	++			1		
94	GGC→AGC	G→S	+				1	
